# Integrative solution structure of PTBP1-IRES complex reveals strong compaction and ordering with residual conformational flexibility

**DOI:** 10.1038/s41467-023-42012-z

**Published:** 2023-10-13

**Authors:** Georg Dorn, Christoph Gmeiner, Tebbe de Vries, Emil Dedic, Mihajlo Novakovic, Fred F. Damberger, Christophe Maris, Esteban Finol, Chris P. Sarnowski, Joachim Kohlbrecher, Timothy J. Welsh, Sreenath Bolisetty, Raffaele Mezzenga, Ruedi Aebersold, Alexander Leitner, Maxim Yulikov, Gunnar Jeschke, Frédéric H.-T. Allain

**Affiliations:** 1 Institute of Biochemistry, Department of Biology, ETH Zürich, Zürich, Switzerland; 2 Laboratory of Physical Chemistry, Department of Chemistry and Applied Biosciences, ETH Zürich, Zürich, Switzerland; 3 Institute of Molecular Systems Biology, Department of Biology, ETH Zürich, Zürich, Switzerland; 4 Laboratory for Neutron Scattering and Imaging, Paul Scherrer Institut, Villigen, Switzerland; 5 Laboratory of Food & Soft Materials, Institute of Food, Nutrition and Health, Department for Health Sciences and Technology, ETH Zürich, Zürich, Switzerland

**Keywords:** Solution-state NMR, Biophysical chemistry, RNA-binding proteins

## Abstract

RNA-binding proteins (RBPs) are crucial regulators of gene expression, often composed of defined domains interspersed with flexible, intrinsically disordered regions. Determining the structure of ribonucleoprotein (RNP) complexes involving such RBPs necessitates integrative structural modeling due to their lack of a single stable state. In this study, we integrate magnetic resonance, mass spectrometry, and small-angle scattering data to determine the solution structure of the polypyrimidine-tract binding protein 1 (PTBP1/hnRNP I) bound to an RNA fragment from the internal ribosome entry site (IRES) of the encephalomyocarditis virus (EMCV). This binding, essential for enhancing the translation of viral RNA, leads to a complex structure that demonstrates RNA and protein compaction, while maintaining pronounced conformational flexibility. Acting as an RNA chaperone, PTBP1 orchestrates the IRES RNA into a few distinct conformations, exposing the RNA stems outward. This conformational diversity is likely common among RNP structures and functionally important. Our approach enables atomic-level characterization of heterogeneous RNP structures.

## Abstract (web summary)

An integrated structural biology approach is utilized to elucidate the solution structure of the polypyrimidine-tract binding protein 1 (PTBP1/hnRNP I) complexed with an internal ribosome entry site (IRES) RNA fragment from encephalomyocarditis virus (EMCV).

## Introduction

Gene expression is critically regulated by protein-RNA interactions. RNA-binding proteins (RBPs) usually contain multiple RNA-binding domains (RBDs), among which the RNA recognition motif (RRM) is the most abundant domain type^1^. Typically, RBDs are flanked and linked by intrinsically disordered regions (IDR) of various lengths and sequences^2^. Recently, these flanking IDRs have been studied extensively in the context of liquid-liquid phase separation^3–5^. For example, the low complexity domain of the ALS-associated protein FUS has not only been connected to functional phase-separated states but is also associated with plaque formation that causes neurotoxicity^6,7^. The IDRs that link RBDs potentially have diverse biological functions and they unavoidably cause flexibility and heterogeneity of the overall protein conformation. However, cooperative binding of the RBDs to RNA-binding sites may lead to an increased conformational order of the protein. This raises questions about the extent to which a disorder-to-order transition is achieved, and whether both flexibility and rigidity are important for the biological function of the protein-RNA complex.

A plethora of structures of isolated RBDs are available from nuclear magnetic resonance (NMR) and X-ray crystallography (see examples in refs. ^8–14^). They generally represent well-defined, three-dimensional folds following the Anfinsen dogma^15^. However, the presence of structurally undefined flexible regions connecting these subunits and the associated conformational heterogeneity hinders the structure determination of full-length RBP and their complexes by using exclusively these classical techniques, as well as cryo-EM^16–18^. Thus, current access to ribonucleoprotein (RNP) structures is limited to protein-RNA machineries with stable intermediates such as ribosomes and spliceosomal particles or to protein-RNA complexes of mainly single and tandem RBDs bound to optimized RNA sequences^19–21^. Yet, the biological function of protein-RNA complexes may depend on the adoption of multiple conformations, which may be important for binding distinct targets^14,22^. Integrative structural biology approaches that combine multiple methods overcome the restrictions of classical techniques in the characterization of such heterogeneous structures^18^. However, integrating a variety of structural constraints with different levels of precision and length, as well as, the aforementioned features of the protein-RNA complexes, also necessitates a shift in the concept of structure determination during the modeling stage.

The abundant 57 kDa RBP polypyrimidine tract protein 1 (PTBP1, also hnRNP I) is a general regulator of cellular mRNA metabolism. In the context of splicing regulation, PTBP1 acts either as a splicing repressor, potentially by binding competition with other factors, or as a splicing activator in a position-specific manner^23–27^. PTBP1 also determines the localization, stabilization and polyadenylation of many target RNAs and is involved in the translational regulation of cellular and viral mRNAs as a prototypical internal ribosome entry site (IRES) trans-acting factor (ITAF)^28^. IRES elements allow non-canonical translation initiation in a 5’-cap independent manner. These large, highly structured RNA sequences are present in the 5’ untranslated regions (UTRs) of particular cellular mRNAs and of the genomes of positive-strand RNA viruses. IRES sequences enable translation during global repression of canonical translation due to cell stress. For instance, during viral infections, canonical translation is arrested and non-canonical translation activates the antiviral response and programmed cell death to prevent further viral spread. In this context, PTBP1 acts as an ITAF and favors the translation of important pro-apoptotic factors^29–31^. Similarly, RNA viruses have evolved PTBP1-responsive IRES elements in their 5’ UTRs to hijack the antiviral response and favor the translation of their viral proteins^32,33^. PTBP1 is among the most frequently found ITAFs and it is an extensively characterized enhancer of the IRES-mediated translation in picornaviruses, including poliovirus (PV), human rhino virus (HRV), hepatitis C virus (HCV), foot and mouth disease virus (FMDV) and several cardioviruses such Theiler’s murine encephalomyelitis virus (TMEV) and encephalomyocarditis virus (EMCV)^34^.

**Fig. 1:**
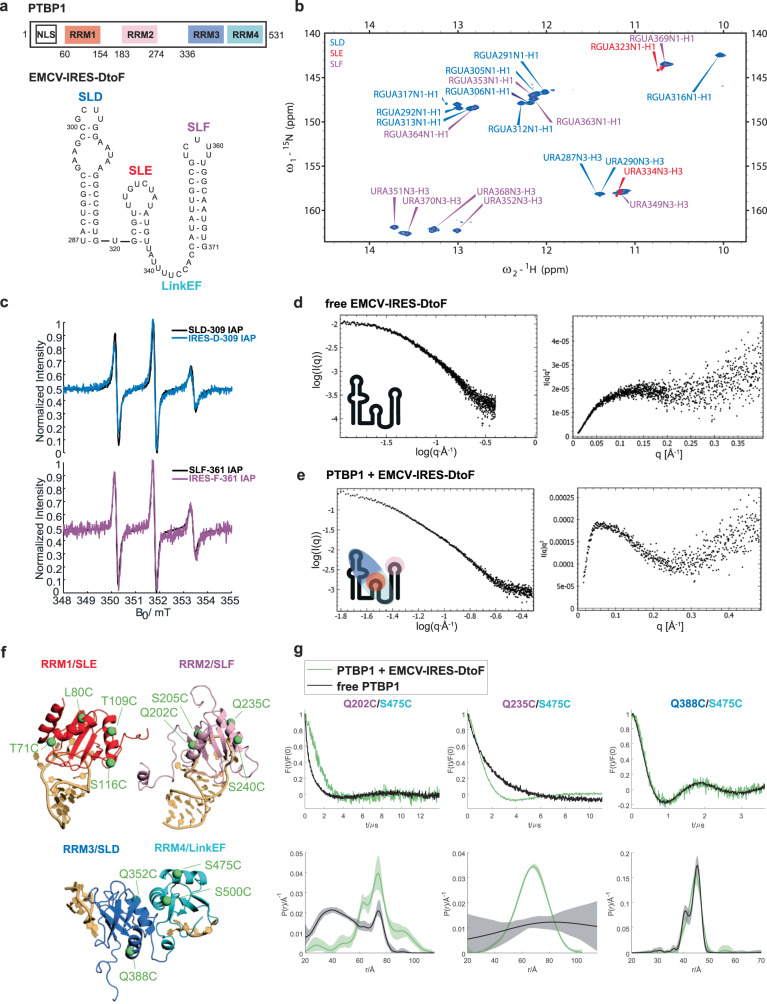
PTBP1 and EMCV-IRES-DtoF are flexible in the free state and rigidify within the complex. **a** Domain scheme of PTBP1 and an overview of the EMCV-IRES-DtoF RNA construct used in this study. **b** 2D ^1^H-^15^N TROSY spectrum of EMCV-IRES-DtoF imino cross-peak signals confirm base-pairing. Imino cross-peaks of G323 and U334 of SLE shifted as indicated by red arrows. **c** CW-EPR spectra of SLD and SLF (black) and EMCV-IRES-DtoF (magenta) with spin label attached to nucleotide 309 and 361, respectively. The sharpness of the signals does not change, implying a similar flexibility of the label in the single SL RNA as in the whole EMCV-IRES-DtoF. **d** The SAXS data measured on free EMCV-IRES-DtoF shows in the Kratky plot (right) the characteristics of a flexible and unstructured molecule. **e** The SAXS curve of the PTBP1-EMCV-IRES-DtoF complex shows in the Kratky plot (right) characteristics of an overall structured molecule with reduced flexibility compared to the free RNA shown in (**d**). **f** Overview of spin-label attachment sites on the individual RRMs of PTBP1 used in this study. Additionally, spin labeling sites were placed in the linker region between RRM1 and RRM2 (152 and 156) and RRM2 and RRM34 (288, 315, 327), respectively. **g** Selected DEER-EPR measurements using spin labels attached to RRM2 (202, 235), RRM3 (388) and RRM4 (475). In the free state (black), signals decay smoothly (top panel), reflecting a broad distance distribution (bottom panel) with the exception of RRM3-RRM4, which interact stably and, thus, lead to an oscillatory decay corresponding to a narrow distance distribution. Upon complex formation (green), the mean distance between RRM2 and RRM4 can increase (202/475) or decrease (235/475), while there is no significant change upon binding for RRM3/RRM4 (388/475). These distance distributions reflect a more ordered and rigidified arrangement of the RNA within the complex. Semi-transparent areas in distance distribution plots correspond to 95% confidence intervals. The data underlying panels (**b**–**e**, **g**) are provided as Source Data.

Structurally, PTBP1 consists of four RRMs, a flexible N-terminus, which contains both nuclear localization and nuclear export signals, and IDR linkers connecting RRM1 with RRM2, as well as, RRM2 with RRM3, respectively^8,35,36^ (Fig. 1a). The nuclear magnetic resonance (NMR) solution structures showed that each of the four RRMs adopts the classical RRM topology of βαββαβ, which is extended by a fifth β-strand in RRM2 and RRM3, and that each domain binds to specific cytosine/uridine-rich sequences within single-stranded RNA^10,37^. It has been proposed that PTBP1 could act as an RNA chaperone since RRM3 and RRM4 stably interact, thereby spatially restricting the orientation of the cognate RNA-binding sites^8,35^. Recent NMR studies of the individual RRM1 and RRM2 bound to UCUUU pentaloops illustrated the capability of PTBP1 to bind to structured RNA targets and identified an α-helix at the C-terminus of RRM1 as of importance for sensing RNA secondary structure^38,39^. However, the interplay of the four domains and their assembly on a natural RNA target remain unknown. Understanding the RNA-binding mode of PTBP1 is important, as misregulation of its expression has been involved in disease promotion, including colorectal cancer invasion, breast cancer cell growth and Parkinson’s disease^40–42^.

Previous studies of PTBP1 in complex with the IRES of EMCV (834 nucleotides, nts) suggested a protein-RNA binding ratio of 2:1 with binding of one molecule of PTBP1 to the IRES stem-loops (SLs) D-F (herein referred to as EMCV-IRES-DtoF, Fig. 1a) and of a second molecule to SLs H-L^43^. Mutation of the RNA interface of the individual RRMs reduced IRES activity for RRM1, RRM3 and RRM4 and abrogated IRES activity for RRM2 and RRM34^44^. Interestingly, mutation of the RNA interface of an individual RRM did not change the binding of the other RRMs. Recently, we revised the interaction pattern of PTBP1 with EMCV-IRES-DtoF and proposed three-dimensional models of the individual PTBP1 RRMs in complex with parts of the RNA^39^.

Here, we applied an integrative structural biology approach combining electron paramagnetic resonance (EPR), NMR, small-angle neutron and X-ray scattering (SANS/SAXS), and cross-linking of segmentally isotope-labeled RNA with tandem mass spectrometry (CLIR-MS/MS), to study the structure of the full-length PTBP1 in complex with an RNA derived from the IRES of EMCV (EMCV-IRES-DtoF, 84 nts). PTBP1 and EMCV-IRES-DtoF are highly dynamic molecules in their isolated states. Upon complex formation, they assemble into a compact and ordered structure while still sampling a fairly large conformational space. This conformational variety can only be described by a structural ensemble. As confirmed using EPR and bicistronic reporter assays, binding of RRM4 to the single-stranded RNA linker between SLE and SLF is crucial for a stable complex assembly as well as for IRES activity, consistent with an RNA chaperone role of PTBP1 in enhancing IRES-mediated translation.

## Results

### PTBP1 and the IRES RNA are flexible molecules in solution

As a first step toward the structural characterization of PTBP1 in complex with EMCV-IRES-DtoF, we analyzed the RNA sequence conservation within cardioviruses (Supplementary Table 1) and the structural conformations of the protein and the RNA in their isolated states using NMR, EPR and SANS/SAXS (Supplementary Fig. 1). The bioinformatics analysis of the EMCV-IRES-DtoF RNA (Supplementary Fig. 1c, d) suggested evolutionary conservation of the stem-loops D and F (herein referred to as SLD and SLF, Fig. 1a), while the stem of stem-loop E (SLE) appeared to be preserved only in EMCV-related viruses. The PTBP1 binding sites for all four domains^39^ in the RNA (pyrimidine-tracts) are also conserved in cardioviruses (Supplementary Fig. 1c, d). Using NMR, the predicted secondary structure of EMCV-IRES-DtoF was validated through the detection of imino proton signals, which report on the base pairing within the RNA SLs. All expected imino protons of stem-loops D, E and F were assigned by assignment transfer from the individual SLs to EMCV-IRES-DtoF (Fig. 1b, Supplementary Fig. 1a, b). The imino proton signals of the individual SLD (except G317) and SLF overlapped perfectly with their corresponding signals in the EMCV-IRES-DtoF RNA, indicating the same fold. Imino proton resonances for the G323-U334 base-pair of SLE were detectable, even though the U334 signal was slightly shifted compared to the isolated SLE that embedded a longer stem (Fig. 1b, Supplementary Fig. 1b). This indicated that the four base-pairs containing stem (321-324/333-336) exists in the EMCV-IRES-DtoF construct, and that, overall, the predicted RNA secondary structure forms in solution, in agreement with its evolutionary conservation (Supplementary Fig. 1c, d). By continuous wave (CW) EPR experiments, we observed that paramagnetic spin labels (iodoacetamido proxyl, IAP) attached to isolated SLs reveal the same EPR line shapes compared to corresponding positions within the entire EMCV-IRES-DtoF, reflecting that the tumbling rate of the spin labels between individual SLs and the full-length RNA construct remained unaffected (Fig. 1c). The small peak-to-peak line widths in these spectra, as well as the nearly symmetric EPR lines, showed that spin labels are in the fast tumbling regime when they are attached to RNA. The spectra are comparable to the previously reported CW-EPR spectra in solution for the individual SLs of the EMCV IRES and exhibit narrower lines than for spin labels attached to single RRMs, which are larger and tumble more slowly^45^. Thus, the spectra indicate substantial relative tumbling of individual SLs with respect to each other in free EMCV IRES. We therefore conclude that in its free state, EMCV-IRES-DtoF is a highly flexible molecule. This conclusion is supported by SAXS measurements recorded on the free EMCV-IRES-DtoF, which resulted in a Kratky plot showing a plateau and no maximum, which is characteristic for highly flexible molecules^46^ (Fig. 1d). The experimental maximal distance D_max_ is 129.00 Å, the radius of gyration (R_g_, see also Supplementary Table 2) is 33.41 Å. This is larger than the value of 24.1 Å calculated according to Hyeon et al.^47^ for folded RNA and thus suggests a structure that is less compacted than a folded RNA with tertiary contacts. Taken together, NMR, EPR and SAXS data show that the RNA in its free form adopts the predicted secondary structure with a large degree of overall conformational flexibility.

Acquiring distance information by EPR requires site-directed spin labeling at two sites within the protein or RNA. For proteins, we exploited methanethiosulfonate spin labeling (MTSSL) or IAP spin labeling and used the four-pulse double electron-electron resonance pulse (DEER) experiment for measuring electron spin-spin distance distributions^45,48^. The inter-domain DEER measurements between spin-labeled sites located in RRM2 and RRM34 revealed broad distance distributions (Fig. 1f, g). This implies the absence of any defined placement of RRM2 with respect to RRM34, consistent with an independent tumbling of RRM2 (as well as RRM1) as noted in previous NMR studies^8^. In contrast, the distance distribution between spin labels attached to RRM3 and RRM4 reflects the well-defined mutual arrangement of the two RRMs by hydrophobic interactions between the α-helices of the RRMs as described earlier^35^. Analysis of the small-angle neutron scattering (SANS) curves suggests an elongated shape for the full-length PTBP1, as already described earlier by others^49^. Interestingly, ensemble modeling of free PTBP1 using EOM^50^ resulted in 14 unique models that (1) were not all extended and (2) did not show any orientational preferences between the rigid bodies RRM1, RRM2 and RRM34. The R_g_ and D_max_ are 46.20 Å and 168.20 Å (Supplementary Table 2).

In summary, the NMR, EPR and SANS/SAXS data show that the free forms of both the RNA and protein are best described by rigid, well-defined bodies (RRMs or SLs) connected by flexible linkers that allow fluctuating spatial arrangements.

### PTBP1 and RNA form a compact complex with residual dynamics

Complex formation between PTBP1 and EMCV-IRES-DtoF causes drastic changes in the structural arrangement of both components. Inter-domain distance distribution measurements by DEER showed clear oscillations in the dipolar evolution functions, corresponding to significantly narrower distance distributions compared to the respective free states (Fig. 1g). This clearly indicates a preferred domain-to-domain orientation within the complex. Considering that all domains bind distinct sites on the RNA^39^, this implies that the RNA structure is also stabilized in a preferred conformation. Importantly, the local secondary structure of the RNA remains unchanged upon PTBP1 binding since the imino proton signals were identical compared to the free state (Supplementary Fig. 1e). In total, we measured a set of 35 DEER distance distributions between 17 spin labeling sites on doubly-labeled PTBP1 in complex with native EMCV-IRES-DtoF (Supplementary Fig. 2, Supplementary Table 3). Interestingly, not all selected spin-label pairs in PTBP1 resulted in narrow distance distributions in the complex, and some spin pairs exhibited standard deviations of distances of up to 28.1 Å. This exceeds the combined conformational flexibility of the two labels of at most 12 Å for MTSSL and 15 Å for IAP by far and thus reflects the preserved flexibility of the complex. Therefore, although the PTBP1/EMCV-IRES-DtoF complex undergoes rigidification and compaction, it also retains certain dynamics and is likely to adopt multiple conformations. In other words, the disorder-to-order transition upon binding is incomplete. Interestingly, DEER measurements between residues located in the RRMs and the protein linkers revealed that the distance distributions between RRM1 or RRM2, and their connecting linker (Link12) are narrower than between RRM2 or RRM3, and Link23 (Supplementary Fig. 2, Supplementary Table 3). Hence, Link12 seems to be more restrained in the complex state and less flexible than Link23. The results from EPR and NMR spectroscopy are in line with SAXS data on PTBP1/EMCV-IRES-DtoF and the free RNA. The experimental R_g_ and D_max_ are 45.03 Å and 138.75 Å, respectively, which are thus smaller than for the unbound protein, indicating compaction. The Kratky plot of the complex compared to the free RNA (Fig. 1e) reflects a more structured molecule^46^ confirming that the IRES RNA, which contributes dominantly to the scattering, is rearranged upon PTBP1 binding.

### Structure determination of the PTBP1-EMCV-IRES-DtoF complex

Due to the substantial flexibility of the PTBP1-EMCV-IRES-DtoF complex, its structure must be represented by an ensemble of conformers. Several approaches have been developed for ensemble modeling based on experimental restraints^50–58^, with some of them specifically geared toward utilizing distance distribution information from EPR^59–65^. Recent hybrid approaches have incorporated experimental data into molecular dynamics simulations to bias the simulations and generate a raw ensemble through enhanced sampling of the relevant regions of conformational space^66,67^. In the subsequent steps of this process, refining the ensemble may involve reweighting to improve the fit of the experimental data to generate the final ensemble^66,68^. These sophisticated approaches draw upon Bayesian statistics to effectively balance the significance of various types of information that may not be entirely consistent. However, given the size and complexity of the PTBP1-EMCV-IRES-DtoF complex, the application of these theoretically well-rooted yet intricate approaches encounters challenges of a technical and computational nature.

Therefore, we have developed a simplified and computationally efficient structure determination protocol that incorporates both short-range distance restraints from NMR and CLIR-MS/MS and inter-domain, long-range distances from EPR and SANS/SAXS to calculate the structure of the PTBP1/EMCV-IRES-DtoF complex. This approach builds upon our previously introduced methodology^60,61^. Here, we essentially defined rigid bodies comprising RRM/RNA sub-complexes, which were positioned with respect to each other by EPR long-range distance restraints using the simulated annealing structure calculation algorithm CYANA^69^. In this step, we used lower and upper bounds for the distances derived from the mean values and standard deviations of the distance distributions reported in Supplementary Table 3. Hence, we also relied on the enhanced biased sampling of the relevant regions of conformational space^58,66^, which, in our case, becomes possible at the level of single conformers by having access to distance distributions rather than only the mean values of distances^62,65^. We note that the standard deviation serves as a measure for the width of the distance distribution, encompassing the combined disorder of the protein backbone and spin-label sidechain. The mean value of the distribution is defined with much higher precision than this standard deviation may imply. The structures of conformers in the initial ensemble were refined by YASARA, and the entire ensemble was fitted against EPR and SAS data (Supplementary Fig. 3). In the subsequent ensemble reweighting step, we utilized the complete distance distribution information, including the shape of the distributions, which may deviate from a Gaussian shape. We accomplished this by maximizing the mean overlap between simulated and experimental distance distributions. This overlap quantifies the shared area between the two distributions. Given that both distributions are normalized to the unit area, this parameter ranges from 0 and 1, where 0 signifies complete disagreement and 1 indicates perfect agreement.

The following observations allowed us to confidently define that the PTBP1/EMCV-IRES-DtoF complex consists of three rigid building blocks connected by flexible peptide and RNA linkers. First, through a combination of NMR and CLIR-MS/MS data for each of the four RRMs of PTBP1, we could place their location to a unique RNA part of the EMCV-IRES-DtoF^39^. The NMR spectra of the full-length PTBP1 bound to EMCV-IRES-DtoF overlap with those of the individual domains bound to their respective RNA targets, suggesting no apparent interactions between the individual domains themselves and with the inter-domain linkers or the N-terminus. This analysis also confirmed that the structures of individual RRMs bound to their RNA targets are preserved in the full-length protein. Second, as a basis for performing site-directed spin labeling (SDSL) and EPR-based distance measurements on the full-length PTBP1 protein, we demonstrated that the EPR-based distance distributions within each isolated RRM are consistent with the NMR-based structures^45^. Importantly, the distance distributions between the labeled positions within each RRM of the full-length protein were identical to those measured on the isolated RRMs (Supplementary Fig. 2). Finally, as shown above, the complex formation does not alter the secondary structure of the EMCV-IRES-DtoF RNA (Supplementary Fig. 1e).

Accordingly, we defined RRM1 bound to SLE, RRM2 bound to SLF and RRM34 bound to SLD (RRM3) and to four nucleotides of the LinkEF (RRM4) as rigid bodies. The structural details of these sub-complexes have been determined previously using a hybrid approach of NMR, CLIR-MS/MS and available structural data^39^. Uncertainties in the RRM1/SLE model were resolved by additional NMR analysis, including the assignment of intermolecular nuclear Overhauser effects (NOEs) to confirm the binding register (Supplementary Fig. 4). Structures of the individual RRMs and SLs were used as input for the structure calculation in CYANA and kept rigid by fixing the backbone torsions within the corresponding segments throughout the calculation. To form RNA stems, we used hydrogen-bond restraints while loops and bulges were maintained flexible. Intermolecular restraints between RRMs and SLs were incorporated to obtain arrangements as in the previously determined structures. In addition to the 35 long-range, protein-protein EPR distance distributions between these three individual sub-complexes, we additionally recorded SAXS data as well as a SANS contrast variation series. The EPR-derived long-range distances were incorporated into CYANA structure calculations as upper and lower limit constraints to position the rigid bodies with respect to each other. Using CYANA, we calculated a raw ensemble of 20,000 models, of which the 2000 models with the lowest target function were selected (for details, see “Methods” and Supplementary Fig. 3). Subsequently, this ensemble was filtered for structural integrity and the individual conformers were refined using YASARA. We refer to the ensemble of 766 conformers obtained at this point as the raw ensemble.

**Fig. 2:**
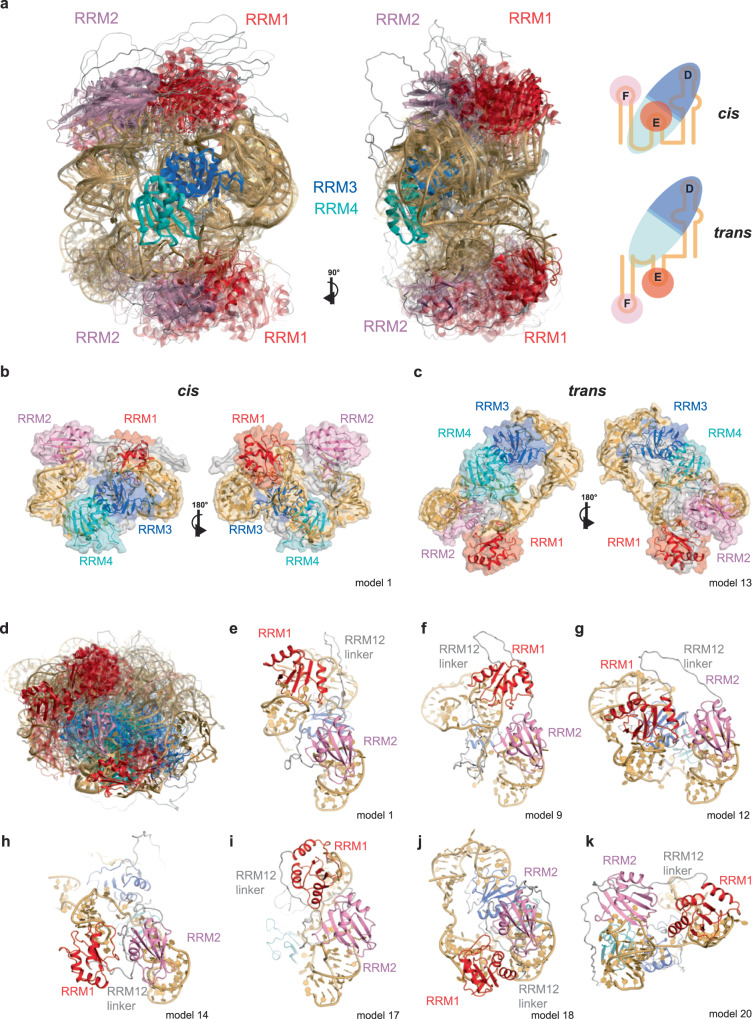
PTBP1 and EMCV-IRES-DtoF form a compacted complex with pronounced conformational flexibility. **a** Structural ensemble of the PTBP1/EMCV-IRES-DtoF complex in a conformer population-weighted visualization (opacity corresponds to the population of each conformer). The 25 models of the ensemble were superimposed on RRM34. Two views related by a vertical rotation of 90° are shown. SLE and SLF can be positioned in *cis* or *trans* with respect to SLD, illustrated in schemes on the right. **b**, **c** Conformers representative of the two subclasses (*cis* and *trans*) in two views. **d** Overlay of the ensemble on RRM2 illustrating the conformational flexibility of RRM1 with respect to RRM2. **e**–**k** Examples of conformers showing different ways RRM1 and RRM2 can be spatially close within the PTBP1/EMCV-IRES-DtoF complex.

To enhance the fit with experimental restraints, we employed integrative ensemble reweighting, where populations are assigned to individual conformers. Simultaneously, we contracted the ensemble by removing conformers with very small populations, while maintaining an accurate representation of the experimental data. This leads to a more concise representation of the ensemble. The process of ensemble reweighting was performed using MMMx^64^, resulting in a final ensemble of 25 conformers (Supplementary Table 4). Each of these conformers is annotated with a distinct probability that ensures a well-balanced fit to the 35 EPR distance distributions between protein sites and the three small-angle scattering curves (Fig. 2a). We refer to this reweighted and contracted ensemble as the main ensemble, and we will elaborate on its structural characteristics in a subsequent paragraph. Altogether, the main ensemble achieves a fit to all the distance distributions, with an overlap ranging between 0.396 and 0.845 for the experimental and back-calculated distance distributions, and a geometric average of 0.662 (Supplementary Fig. 5). A loss of merit of 0.332 upon integrating distance distribution and small-angle scattering restraints indicates that both sets of restraints are largely, but not entirely, consistent with each other (Supplementary Figs. 3 and 6a–f). The good agreement of the back-calculated SAXS and SANS curves in the small-angle range demonstrates the accuracy of our model in fitting the size of the complex.

We conducted a series of tests to assess the robustness of the main ensemble. In the first test, we excluded the 25 conformers of the main ensemble from the raw ensemble and then repeated the ensemble reweighting and contraction with only the remaining 741 conformers. The resulting validation ensemble comprises 30 conformers and demonstrates a smaller loss of merit (0.266) compared to the main ensemble. Furthermore, a geometric average distance distribution overlap of 0.683 is achieved, slightly surpassing that of the main ensemble (Supplementary Fig. 7a, Supplementary Table 5). Among the small-angle scattering curves, the fit quality for the 4 m SANS curve slightly improves, while there is a slight decline in fit quality for the SAXS curve and a moderate decline for the 1.2 m SANS curve. When all conformers are superimposed on RRM34, pseudo-electron densities of the main and this validation ensembles exhibit 79% overlap with each other (Supplementary Table 6).

For the second test, we implemented a new ensemble reweighting algorithm into MMMx. With this algorithm, we fitted the populations of all 761 conformers in the raw ensemble simultaneously by non-negative linear least squares (NNLLSQ ensemble). After the fitting process, we eliminated conformers with less than 0.1% of the total population, resulting in an ensemble composed of 32 conformers. This approach involved fitting to background-corrected DEER traces rather than distance distributions and utilizing least-squares instead of minimizing *χ*^2^ for the small-angle scattering curves. Nevertheless, we assessed the fit quality using the same figures of merit employed for the main ensemble (Supplementary Table 5). The pseudo-electron density of the NNLLSQ ensemble exhibits 73% overlap with the main ensemble (Supplementary Table 6). The NNLLSQ ensemble is visualized in Supplementary Fig. 7b.

In a third test, we maximized distance distribution overlap while ignoring the small-angle scattering curves. This DEER-only ensemble, visualized in Supplementary Fig. 7c, features 62 conformers. Its pseudo-electron density exhibits 68% overlap with the main ensemble (Supplementary Table 6).

Comparison of the fit quality of all these ensembles with the raw ensemble (Supplementary Table 5) reveals that ensemble reweighting and contraction generally improve figures of merit. The raw ensemble provides already a good overlap with the distance distributions, as the distance restraints were considered in its generation, but very poor agreement with the small-angle scattering curves. This latter agreement improves substantially in the main and validation ensemble, albeit at the expense of some deterioration of overlap with the distance distributions. This suggests a certain level of inconsistency between DEER data and small-angle scattering curves, which might be caused by the requirement to perform DEER measurements under cryogenic conditions. Below, we will discuss the features of the main ensemble, keeping in mind the uncertainty implied by the variation between the main, validation, and NNLLSQ ensembles.

In a final validation fit, we computed a model of only the protein in the context of the complex by ignoring topological restraints imposed by the RNA. In this, we could still use the SANS curves, since the solvent matches RNA neutron scattering, as well as the EPR distance restraints, since all our labeling sites are on PTBP1. We generated the raw ensemble by the RigiFlex approach as implemented in MMMx^64^ and performed ensemble reweighting as for the main and validation ensembles, except for skipping the SAXS curve. This protein-only ensemble is visualized in Supplementary Fig. 8a. While the protein-only ensemble cannot be compared quantitatively with the other ensembles, it reveals that the general arrangement of the RRMs, including the presence of the cis and trans sub-ensembles discussed below, is encoded by the DEER restraints and the SANS curves alone. Indeed, this arrangement even persists when skipping the SANS curves. This finding implies that the RRM arrangement found in the present study does not depend on the previous CLIR-MS/MS results^39^, which are, however, required for modeling the RNA.

Finally, we tested how the fit quality and size of the reweighted ensemble depend on the size of the raw ensemble (Supplementary Fig. 9). We observed that the overlap of experimental and back-calculated distance distributions reasonably converges for raw ensembles with a size of more than 600 conformers. The mean *χ*^2^ of the three small-angle scattering curves is also converged for this size of the raw ensemble, whereas the size of the reweighted ensemble varies slightly (21 to 26 conformers).

As seen in Supplementary Fig. 5, the overlap between experimental and fitted distance distributions is generally smaller than expected from only the uncertainty of the experimental distributions. For these broad distributions, additional uncertainty from spin-label rotamer simulations is expected to be a minor contribution. In particular, some of the simulated distributions are clearly bimodal, reflecting the cis and trans sub-ensembles discussed below, whereas the corresponding experimental distributions are so broad that bimodality cannot be resolved. To some extent, this is reflected in the broader conformer distribution of the DEER-only ensemble (Supplementary Fig. 7c). However, even this ensemble exhibits bimodality in conformer distribution. This bimodality is more pronounced in the integrative model that also considers the small-angle scattering data, which may indicate that the shock-freezing required for the DEER measurements affects conformer distribution to some extent. For individual data sets with particularly poor overlap, such as 205/388 and 235/388, we cannot safely exclude that the label itself biases the conformer distribution. However, in the DEER-only ensemble, overlap improves from 0.397 to 0.692 for the pair 205/388 and from 0.449 to 0.605 for the pair 235/388, indicating that inconsistency between distance distributions and small-angle scattering data is the main reason for the poor fit of these distributions by the main ensemble. In the following, we discuss structural features of the main ensemble that are reasonably robust with respect to fit procedure and restraints used.

### A structure with several conformations exposing the RNA

The pairwise root mean square distance (rmsd) between the 25 conformers of the PTBP1/EMCV-IRES-DtoF main ensemble varies from 2 to 30 Å over the entire complex (Supplementary Fig. 6g, h), reflecting the wide distance distributions measured for many spin pairs. Despite this apparent structural heterogeneity, the structural ensemble of the complex reveals very interesting features. The best way to illustrate the conformational space covered by this structural ensemble is to superimpose each conformer onto RRM34 (Fig. 2a). This representation reveals that RRM1 and RRM2 localize primarily on two sides with respect to RRM34. Based on the position of RRM1 and RRM2 relative to RRM34, the structural ensemble can be divided into two groups with a major form (13/25 conformers, with a 60 ± 3% population by amounting the individual probabilities) and a minor form (12/25 conformers, with a 40 ± 3% population). In the major form, SLF and SLE are in cis with respect to SLD (Fig. 2b), while in the minor form, the two SLs are in trans with respect to SLD (Fig. 2c). In the cis conformation, RRM1 and RRM3 are close to each other, while in the trans conformation, they are more distant. Importantly, despite being separated by a flexible linker, RRM1 and RRM2 are always within close proximity. Yet, RRM1 is not situated at a fixed position relative to RRM2 but occupies multiple sites with distinct orientations (Fig. 2d). Some of the determined RRM1 and RRM2 orientations can be explained energetically by the conformation of the inter-domain linker, molecular contacts involving the domains and the linker or the two domains and protein-RNA intermolecular interactions (see below) (Fig. 2e–k). Independent structural ensembles generated by either removal of the 25 conformers that were included in the best-fitted integrative ensemble (Supplementary Fig. 7a) or fitting only against DEER data (Supplementary Fig. 7c), resulted overall in similar conformational ensembles composed of these two cis/trans families.

PTBP1 RRM34 interacts with EMCV-IRES-DtoF using the same interface and, remarkably, the same directionality, which we had previously identified when studying its binding to single-stranded RNA^70^. Indeed, RRM3 binds the RNA at the 5’-end (SLD) and RRM4 further downstream (LinkEF) using the interaction surface near the small helix of the linker. This is seen in both the major and minor forms of the complex. However, only in the minor form, the trans arrangement brings the intervening RNA near the RRM34 inter-domain linker enabling additional protein-RNA interactions (Fig. 2c). As independent support for these contacts, we could identify protein-RNA cross-links between this region of the linker and the RNA (Supplementary Fig. 10). The detection of these protein-RNA cross-links to U/UU validate the contacts found in this subpopulation of conformers (Supplementary Fig. 10e). Strikingly, hydroxyl radical cleavage data^43^ for the linker residue N432 support its position close to SLE (Supplementary Fig. 11). The two main conformations originate from RRM4 binding to the linker between SLE and SLF, thereby restricting the mobility of the SLs, but still allowing SLD and SLE to be positioned in cis and trans as not all nucleotides of the LinkEF sequence are bound by RRM4. In the trans conformation, the RRM34 inter-domain linker seems to contribute to the stabilization of this form. Additional “weak contacts” between RRM1 and RRM2 then help further stabilize most conformers of the complex (see below).

Importantly, despite the very strong compaction seen upon the complex formation of the RNA and the protein (Supplementary Fig. 8b), we still observe residual conformational flexibility (Fig. 2a). As indicated above, an unexpected and interesting feature of the PTBP1/EMCV-IRES-DtoF complex is the spatial proximity of RRM1 and RRM2, which is induced upon RNA binding. RRM1 and RRM2 do not strongly interact, resulting in RRM1 being in proximity to RRM2 but not at a fixed position (Fig. 2d). This proximity is facilitated by the 10 residues C-terminal to RRM1 that fold into an α3-helix upon binding to SLE, reducing the overall length and dynamics of the linker^38^. Although the DEER data is not sufficiently resolved to discuss features at atomic length scales, and we have only one distance distribution restraint corresponding to the linker between RRM1 and RRM2, the entropy of this linker is somewhat reduced by the relative arrangement of these two RRMs. However, we cannot rule out the possibility that the following observations are influenced by the refinement of the conformer structures in YASARA using the AMBER force field. Close examination of the 25 conformers revealed that RRM1 may favor conformations placing it close to RRM2 because the inter-domain linker is very hydrophobic and folds back as a hairpin, resulting in RRM1 facing the β-sheet of RRM2 (seen in models 1–7) (Fig. 2e). In other cases (models 11-12) (Fig. 2g), contacts between the α3-helix of RRM1 and the inter-domain linker between RRM2 and RRM3 brings RRM1 close to RRM2. In another subset (models 8-10), the α2-β4 loop of RRM1 interacts with the β3-α2 loop of RRM2, and the inter-domain linker makes contacts with the α1-α2 surface of RRM1, resulting in the β-sheets of both RRMs facing in the same direction (Fig. 2f). In a fourth subset (models 13-15), the RRM1-RRM2 proximity is mediated by the RNA, since SLE is sandwiched between both RRMs (Fig. 2h). In a fifth subset (models 16-17), α2 of RRM1 mediates contacts to the top part of RRM2 (Fig. 2i) and in one case (model 18), the two domains are arranged back-to-back (Fig. 2j). In a final subset (models 20-25), the two domains do not form any contacts and the inter-domain linker is elongated interacting with the RRMs (Fig. 2k). This illustrates the complexity of the conformational landscape of this type of protein-RNA complex, where weak interactions between folded domains, protein linkers and RNA are competing, resulting in a globally compact but still very dynamic structural ensemble.

Overall, a key aspect of this PTBP1-IRES structural ensemble is that protein binding results in a compacted RNP structure with reduced (compared to the free protein and RNA), but still pronounced conformational flexibility. Importantly, the four RRMs lie in the interior of the structure and expose the three conserved RNA stems to the outside (Fig. 2a–c). This conformation could explain the proposed chaperone role of PTBP1, since exposing these RNA stems presents the IRES structure for subsequent interactions with the ribosome or translation initiation factors as seen in IRES-ribosome structures^71–74^ (see “Discussion”).

### RRM4-LinkEF interaction is crucial for IRES activity

**Fig. 3:**
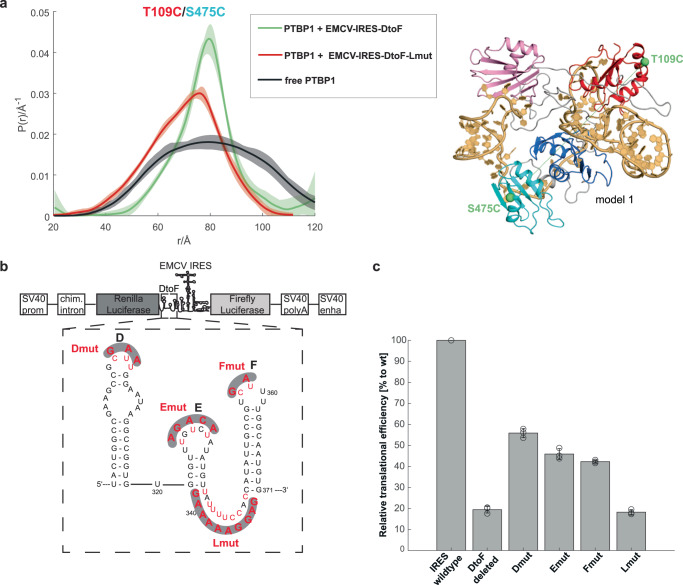
RRM4 binding is crucial for complex stabilization and translation enhancement. **a** Removal of the pyrimidine stretch in LinkEF (same mutation as shown in (**b**) for the Lmut Luciferase Reporter Assay construct) interferes with RNA binding of RRM4 and broadens the distance distribution measured between spin labels attached to RRM1 and RRM34 significantly. Compared to free PTBP1, the distance distribution narrows upon binding of EMCV-IRES-DtoF, but the mean distance does not shift substantially. This effect is less pronounced with the Lmut. Semi-transparent areas in distance distribution plots correspond to 95% confidence intervals. **b** Constructs for the Luciferase Reporter Assay (top) with the deleted or mutated (pyrimidine to purine) sequence of EMCV-IRES-DtoF (bottom, only one mutation at a time). **c** Luciferase Reporter Assay given as IRES-mediated translation efficiency with respect to the WT-IRES. Remarkably, the removal of the RRM4-binding site (Lmut) results in the same activity loss as the deletion of the whole EMCV-IRES-DtoF sequence, which links the importance of RRM4 binding for complex stabilization and rigidification with translational efficiency of the IRES. The error bars represent the standard deviations of the three biological replicates. The value for each biological replicate was determined as a mean of three technical replicates. The data underlying panels (**a**, **c**) are provided as Source Data.

The RRM4-LinkEF interaction is central to the complex structure as it stabilizes the longest RNA linker (LinkEF) and thereby participates in the compaction of the complex. We therefore investigated the effect on the structural integrity of the complex by interfering with the RRM4-LinkEF interaction to test its functional importance (Fig. 3). Replacement of the pyrimidine tract in LinkEF with purines (Lmut in Fig. 3b) resulted in a complex with significantly broadened distance distribution between spin labels on RRM1 and RRM34 compared to the non-mutated complex (Fig. 3a), while the RNA secondary structure remained unchanged (Supplementary Fig. 1f). Similarly, mutation of key amino acids for RNA-binding of RRM4 to alanine (H457A, R523A, K528A; referred to as RRM4ko) resulted in broader distance distributions between RRM4 and RRM2, and between RRM1 and RRM2, compared to non-mutated PTBP1 (Supplementary Fig. 12). These experiments confirm that RRM4 is crucial for the stabilization of the complex.

To explore if altering the binding of the different RRMs would affect the biological functionality, we investigated the influence of each individual RRM-binding site on IRES activity using a bicistronic luciferase reporter assay in HEK-293T cells^75^. In this assay, Renilla Luciferase is translated 5’-cap-dependent and Firefly Luciferase is translated under the control of the EMCV-IRES, both from the same transcript. Thus, the ratio of Firefly Luciferase activity to Renilla Luciferase activity is a direct measure of IRES activity^76^. We first compared the activity of the EMCV-IRES wild-type (WT) (Fig. 3b, c) with a deletion mutant that lacks the entire EMCV-IRES-DtoF sequence (“DtoF deleted”). The deletion showed about a fifth of the WT activity (down to 19 ± 1.0%), in line with previous reports that this part of the IRES is not essential but important for stimulating the IRES activity^33^. Mutating the individual RRM-binding sites on SLD, SLE or SLF by exchange of pyrimidines to purines led to a strong reduction down to 56 ± 2.7%, 46 ± 1.8% or 42 ± 1.8%, respectively (Fig. 3b, c). Strikingly, mutating pyrimidines in the RRM4-binding site (LinkEF) to purines reduced the activity to 18 ± 0.4%, similar to the deletion of the complete EMCV-IRES-DtoF. This strongly suggests that structural stabilization and compaction by RRM4 is crucial for EMCV-IRES activity.

Together these experiments revealed rather surprisingly that a flexible RNA linker, which connects two SLs, has an essential role in the biological activity of an RNA molecule. PTBP1 binds to the flexible RNA linker and remodels the overall RNA structure. The resulting structure and the ordering of the RNA structure upon PTBP1 binding are crucial for the IRES-mediated translation initiation, potentially by facilitating the recruitment of the ribosome or interaction with translation initiation factors^28,72–74^. The overall conformation that exposes the RNA stems and the remaining conformational dynamics may allow the RNP to present a subset of conformations that can be recognized by the translation machinery through conformational capture. In contrast, without PTBP1 binding, the inherent flexibility due to the lack of linker binding loosens the complex arrangement and therefore impedes translation initiation.

## Discussion

Here, we present insights into the relationship between structure, dynamics and function of a multi-RBD protein-RNA complex by modeling its ensemble structure, which can lead to multimodal function. Since we took an approach to determine the structure, which integrates five structural methods and aims for the best fit among the different structural constraints, two questions may arise: How accurate is the structural ensemble, and what does it tell us about the biological function? Reassuringly, the presented structural ensemble is consistent with the hydroxyl radical cleavage that was performed by others^43^, and thus this data can be seen as an independent support for the structural conformers (Supplementary Fig. 11). Additionally, the identification of the trans conformation helped us explain, retrospectively, the cross-links we identified earlier^39^ between the linker of RRM34 and the RNA (Supplementary Fig. 10). Functionally, our results rationalize the proposed roles of PTBP1 as RNA chaperone and ITAF in the context of IRES-mediated translation initiation. In recent years, a few structures of IRES elements bound to ribosomes have been determined^71–74,77^, however, in all these cases, the IRES RNA is stabilized exclusively by pseudoknots and RNA tertiary interactions, although most viral and cellular IRESs require protein ITAFs for optimal translation activity. Our structural work provides evidence that PTBP1 strongly compacts the RNA and stabilizes the RNA fold into discrete conformations. As a result of PTBP1 binding, the RNA helices of SLD, E and F are exposed to the solvent for further interactions with the translation machinery, consistent with what was found in protein-free IRESs bound to ribosomes^71–74,77^. Interestingly, in particular, the sequence of the solvent-exposed SLD containing a purine-rich internal loop is conserved, suggesting that the structure and the sequence of the exposed IRES element may be recognized by the ribosome or additional factors (Supplementary Fig. 1). In the context of EMCV-IRES, the SL J-K are bound by the HEAT-1 domain of eukaryotic initiation factor 4G (eIF4G), which require a pre-organization to position protein-recognition^78^. Interestingly, a second PTBP1 molecule has been shown to bind to this region of the EMCV-IRES and thereby may contribute to this organization, which has been shown to be common in type 2 picornavirus IRESs^43,79^. The compaction and stabilization effect was not necessarily expected because PTBP1 itself is a very dynamic protein containing four folded domains separated by flexible linkers, and previous studies on the shape of the free protein have established the view that PTBP1 is present in solution as an elongated particle^36,49^. A number of features unique to PTBP1 make the protein able to fold EMCV-IRES-DtoF into a structured RNA: First, the four RRMs separated by flexible linkers allow binding to separate RNA sequences within the IRES. The observed RNA structure stabilization through PTBP1 binding explains also previous data showing that mutations of individual RRMs do not disrupt RNA binding, but still result in a strong reduction of translation efficiency^44^. Second, the tight interaction between RRM3 and RRM4 brings distant RNA sequences into close proximity. Our structural and functional study identifies the interaction of RRM4/LinkEF as a major element for structuring the core of PTBP1/EMCV-IRES-DtoF. An increase in flexibility of the complex upon mutation of the RRM4-binding site in LinkEF coincides with a dramatic reduction in translation efficiency. Third, the binding of RRM1 to SLE results in a shortening of the linker between RRM1 and RRM2^38^. Fourth, there is a close interaction between RRM1 and RRM2 that is mediated by several sets of interactions and not a unique one. This last feature is primarily responsible for the residual conformational flexibility seen in the final structural ensemble. Conformational flexibility was also seen in protein-free IRES structures^71–74,77^, so it seems to be a common structural feature of IRESs. This flexibility may help with the initial recognition by the ribosome and also for the IRES to adapt its structure during the different phases of translation that are accompanied by the structural dynamics of the ribosome. In agreement with this, recent in vivo single-molecule experiments showed that EMCV-IRES transitions between translationally active and inactive RNA states^80^. Such RNA remodeling capability of PTBP1 is also likely to be important for splicing regulation, consistent with reports supporting the role of RRM4, and the interaction with RRM3 for splicing activity^70,81–83^. Thus, compaction and stabilization of the RNP complex is not restricted to IRES-mediated translation initiation, but a general mode of action of PTBP1.

The structural details presented here also provide general insights into how RBPs interact with RNAs. To our knowledge, the only other structure of a multi‐RRM containing protein with more than two RRMs in complex with a natural RNA is the crystal structure of the spliceosome-specific RNA chaperone Prp24 bound to U6 snRNA^84^. Both Prp24 and the U6 snRNA experience large structural rearrangements upon complex formation, and the crystal structure has a rigid interlocked topology. In this case, only three of the four RRMs interact with RNA (RRM2-4), and RRM2 interacts extensively with RRM1 and RRM4, while the inter-domain linker between RRM3 and RRM4 folds into two α-helices upon RNA binding. Therefore, PTBP1 and Prp24 use completely different modes of action, although both act as RNA chaperones. Importantly, while Prp24 has a single RNA-binding substrate, PTBP1 has thousands of RNA-binding targets being involved in almost all post-transcriptional gene regulatory processes^85^. Therefore, it may not be surprising that the complex formation of PTBP1 results in a discrete number of complex structures and not a single one. Moreover, it is likely that it would be energetically more favorable for an RNP complex to have a higher degree of flexibility and thereby maintain a higher conformational entropy compared to a rigid structure, especially considering the large entropic cost associated with the binding between two very flexible molecules. The preservation of conformational dynamics in the bound state may also facilitate the recruitment of additional binding partners and allow them to exert additional influence on the RNAs conformational landscape, required to attain functional conformations.

We expect that the approach presented here will prove valuable for future structural characterizations of RNP complexes involving the numerous RBPs in the human genome that, similarly to PTBP1, are composed of multiple RBDs separated by flexible linkers. RNA binding may result in several, substantially different conformations and could be vital to their function. Many RNP machineries rely on major structural rearrangements, as seen in the different steps catalyzed by the spliceosome or the ribosome^19,20^. Our approach that combines short and long-range distance constraints from NMR, MS and SAS, as well as, distance distributions from EPR, allows us to determine a structural ensemble covering the full conformational space covered by such dynamic RNPs. Distance distributions from EPR experiments inform on the width of conformational distributions and thus represent a basis for integrative structure modeling of large systems, provided that three-dimensional models of the structurally well-defined building blocks already exist. The recent advances in AI-driven structure predictions will aid the availability of such building blocks^86^. Further work is required in perfecting integrative ensemble modeling and, in particular, in providing uncertainty estimates for broad ensembles. Integrating NMR and EPR data, protein-RNA cross-linking restraints, and SAS curves holds much promise as a strategy to determine ensembles of rigid-body arrangements and even protein-RNA condensates, to provide insights into the relationship between structure, dynamics and function of RBPs.

## Methods

### Site-directed mutagenesis

Positions for site-directed spin labeling (SDSL) on the protein were chosen based on simulating the spin-label attachment to existing NMR solution structures of individual RRMs of PTBP1^8^ using the software package Multiscale Modeling of Macromolecular systems (MMM)^87^. For inter-domain EPR distance measurements, we chose amino acids in the RRMs that are predominantly located within the α-helices, with geometrical similarities and related chemical properties compared to cysteine. Positions in the peptide linker regions were selected close to the C-terminus of RRM1 and RRM2, close to the N-terminus of RRM3 or in the center of the linker. Site-directed mutagenesis was performed using quick-change PCR with specific primers listed in Supplementary Table 7 and pET28a-PTBP1(1-531) plasmid reported earlier as template. Cys250 and Cys251 were mutated to serines in all constructs. Cys23 was mutated to serine in all protein constructs for EPR measurements.

### Protein expression and purification

Protein expression and purification of PTBP1 and individual RRMs were performed as described previously^39,45^. For distance measurements >5.5–6 nm, expression was performed in 95–99% D_2_O using minimal M9 medium followed by the same purification protocol. Samples for NMR and SANS experiments were finally transferred to 10 mM NaPO_4_, pH 6.5, 20 mM NaCl buffer with 1–2 mM DTT by size-exclusion chromatography and dialysis.

### RNA preparation

In vitro transcription of EMCV-IRES-DtoF and of individual SLs was performed as described previously^39,45^. For spin labeling of RNA, respective RNA segments were removed by RNase H cleavage and chemically synthesized (Dharmacon) thio-uridinated RNA segments were ligated after spin labeling similarly as described before^45,60,88^.

### Site-directed spin labeling of protein and RNA

Spin labeling protocols used for all PTBP1 mutants have been described elsewhere^45^. For SDSL of PTBP1, we used predominantly MTSSL ((1-oxyl-2,2,5,5-tetramethylpyrroline-3-methyl) methanethiosulfonate, Toronto Research Chemicals) or IAP (3-(2-iodoacetamido)-proxyl, Sigma Aldrich). For RNA SDSL, different uridine positions in the loop or linker regions in EMCV-IRES-DtoF were selected and the respective short oligonucleotides containing 4-thiouridine were spin-labeled and ligated to the full-length RNA following earlier published protocols^45,60^.

### PTBP1/EMCV-IRES-DtoF complex formation

Complex formation and purification were performed essentially as described before^39^. In brief, EMCV-IRES-DtoF was diluted in all cases to approximately 4 µM in 5 mL low salt buffer (10 mM NaPO_4_, 20 mM NaCl, pH 6.5). Concentrated doubly-labeled PTBP1 was mixed with the RNA in a molar ratio of 1:1.2 and the complex was then purified by size-exclusion chromatography using a Superdex 75 column (Cytiva). Complex samples were then buffer-exchanged into D_2_O and concentrated to approximately 50–100 µM. For DEER distance measurements, complex samples were diluted 1:1 (v/v) with d_8_-glycerol (Sigma Aldrich), and 30 µL sample solution was filled into 3 mm quartz tubes. Complex formation was ensured by native RNA polyacrylamide gels.

### Bicistronic reporter assays

Bicistronic pRemcvF plasmids were a generous gift from Prof. Dr. A. Willis (MRC Toxicology Unit, Leicester, UK)^89^. EMCV-IRES mutants were generated using standard site-directed mutagenesis procedures. HEK-293T cells were derived from a lab stock and maintained in Dulbecco’s modified Eagle’s medium supplemented with 10% heat-inactivated fetal calf serum (GibcoBRL) and antibiotics. For IRES activity assays, a 24-well plate was seeded with 500 µL cell suspension (approx. 350,000 cells/mL). After 24 h growth, the medium was exchanged to medium without antibiotics. Then, cells were transfected with 500 ng plasmid/well and a final volume of Lipofectamin 2000 (Thermo Fisher) of 1 µL/well in a total volume of 100 µL OPTI-MEM (Gibco) medium/well. Cells were incubated for 24 h and lysed using the Dual-Luciferase Assay lysis buffer supplemented with Roche Complete Protease Inhibitor (Roche, incubation of 10 min on a shaker). Lysates were cleared by centrifugation (16,000 × *g*, 4 °C). Then, 5 µL of lysate/well was pipetted into a 96-well ELISA plate and 25 µL of Firefly-Luciferase substrate solution was added and measured after 15 s shaking in the plate reader. Then, 25 µL of Stop-N-Go solution was added and samples were measured to assess the Renilla-Luciferase activity. All IRES assays were performed in technical triplicates of biological triplicates and measured on a Berthold MicrolumatPlus luminometer. IRES activity is determined by normalizing the Firefly-Luciferase activity to the Renilla-Luciferase activity, whereas the activity of the WT-plasmid is set to 100%.

### CLIR-MS/MS

Mass spectrometry data from previous CLIR-MS/MS analysis of the PTBP1-IRES complex^39^ was reanalyzed with an optimized parameter set containing more neutral loss products, to increase the number of identified protein-RNA cross-links. Mass spectrometry data files (Thermo Fisher RAW format) corresponding to the uniformly labeled EMCV-IRES RNA experiment were retrieved from ProteomeXchange via the PRIDE partner repository (PXD005566) and converted to mzXML using msconvert.exe (ProteoWizard msConvert v.3.0.9393c)^90^. The files were searched using RNxQuest^91^, an add-on to xQuest (both available for download from https://gitlab.ethz.ch/leitner_lab/)^92,93^, against a database containing only the target PTBP1 protein sequence, with expected light-heavy RNA adducts defined as mono-links. Adducts with lengths of 1–4 nucleotides were considered. In addition to the whole RNA adduct, the following neutral losses were considered: -H_2_O, -H_4_O, -HPO_3_, -H_3_PO_3_, -H_2_, -HPO_3_+H_2_O, -HPO_2_, -H_4_O_2_, -H_5_PO_5_. Further details about the expanded parameter set can be found elsewhere^94^. All amino acids were set as cross-linkable. Delta masses for each adduct were defined depending on the nucleotide sequence composition, as described previously^39^. Further parameters used for xQuest searching: delta mass tolerance: +/− 15 ppm, retention time tolerance: 60 s, enzyme = Trypsin, maximum missed cleavages = 2, MS1 mass tolerance = 10 ppm, MS2 mass tolerance = 0.2 Da. Identifications with an ld.Score > 20 (according to the scoring scheme described previously^92^) were considered. Further processing and visualization were performed using the scripts contained in the RNxQuest package. The reanalyzed mass spectrometry proteomics data have been deposited to the ProteomeXchange Consortium (http://proteomecentral.proteomexchange.org) via the PRIDE partner repository^95^ with the dataset identifier PXD034894.

### NMR spectroscopy

All NMR experiments were recorded on Bruker Avance III 500, 600, 700 or 900 MHz spectrometers equipped with cryo-probes and on a Bruker Avance III 750 MHz spectrometer with a room temperature probe. NMR protein spectra (free proteins and protein-RNA complexes) were acquired at 313 and 323 K, imino ^1^H^1^H-NOESY spectra were recorded at 278 or 283 K. Imino-^1^H^15^N-TROSY spectra were recorded at 278 and 293 K. We processed spectra with Topspin 2.1 or Topspin 3.0 and analyzed in Sparky 3.0^96^. ^1^H-^1^H imino assignments of the RNA were achieved by standard methods^97,98^. Imino resonances of SLE were assigned using conventional jump-return NOESY with 200 ms mixing time and facilitated with SMT experiments^99^ at 278 and 275 K. Non-exchangeable protons were assigned using sequential walk in NOESY spectra recorded at 298 K and in 100% D_2_O NMR buffer (10 mM NaPO_4_, pH 6.5, 20 mM NaCl buffer with 1 mM DTT). Assignment of other hydrogens and heteronuclei was obtained using intra-nucleotide assignment experiments, including aromatic HSQC, CT HSQC of sugar resonances, TROSY HCCH COSY for adenine H2-H8 assignment, HCCH COSY for H5-H6 correlations of pyrimidines, and finally HCN and HCNCH experiments. Assignments of protein resonances were obtained using conventional triple resonance experiments, HCCH-TOCSY and 3D NOESY-HSQCs. Intermolecular NOEs were identified in ^13^C,F3-filtered HSQC-NOESY experiments using 100–150 ms NOE mixing time and L-PROSY NOESY recorded with 10 loops and 40 ms per loop^100,101^. Throughout the study, simple titration experiments were recorded using 0.2 mM protein/RNA samples, assignments experiments for molecules in the free state were performed with 1 mM samples, and the protein-RNA complexes were at approximately 0.7–0.8 mM of each molecule.

### EPR spectroscopy

Labeling efficiencies and spin-label tumbling regimes of spin-labeled protein and RNA samples were determined by CW-EPR spectroscopy at ambient temperature. Experiments were performed at X band (9.5 GHz) using a Bruker Elexsys E500 spectrometer with a Bruker super-high Q resonator ER4122SHQ and spectra were recorded with a field modulation of 100 kHz, a modulation amplitude of 1 G, a time constant of 10.24 ms, a conversion time of 40.96 ms and an attenuation of 25 dB of 200 mW incident microwave power. Samples were filled into glass capillaries (BLAUBRAND®) with a diameter of 1.5/0.9 mm (outer/inner). Labeling efficiencies were determined by digital double integration of the EPR spectra and comparing them to free nitroxide radical IAP of standard concentration. Changes in the spin-label mobility could be detected by amplitude reduction of the low- and high-field components of the nitroxide spectra, which indicated successful spin labeling.

To obtain EPR distance restraints, four-pulse DEER experiments^48^ were performed at a home-built high-power Q-band spectrometer (35 GHz) with 200 W microwave power, a Bruker ElexSys acquisition system (E580) and a home-built TE001 pulse probe^102^. Temperature stabilization during all measurements was ensured by a He-flow cryostat (ER 4118CF, Oxford Instruments) and a temperature control system (ITC 503, Oxford Instruments). All free protein and complex measurements were carried out at 50 K because this temperature corresponds to the optimal conditions for nitroxide radicals with respect to their longitudinal and transverse relaxation. Pump pulses were always set to 12 ns and detection pulses to 16 ns. While a setup with all 12 ns pulses may provide better sensitivity, it requires a very good resonator mode and thus careful sample volume and sample position adjustment. In practice, we found it more convenient to slightly compromise the bandwidth of the detection pulses by increasing their length from 12 ns to 16 ns. The first inter-pulse delay τ_1_ was set to 400 ns, τ_2_ was set according to the expected distance and its required DEER trace length for suitable background correction. Time increment *t* was also adapted to the length of the τ_2_ as described earlier^45^. The pump pulse was always applied on the maximum field position of the nitroxide spectrum, whereas detection pulses were applied by an offset of approximately 100 MHz. Samples that were diluted 1:1 (v/v) with d_8_-glycerol (Sigma Aldrich) were shock-frozen with liquid nitrogen and measured in this state.

### Analysis of DEER data

For processing all DEER data we used the software package DeerAnalysis^103^, version 2022, in automated mode. When an artifact arose from the overlap between the excitation bands of the pump and detection pulses^103,104^, up to 15% of the data was cut at the end. For background correction, we used a dimensionality of 3, corresponding to a homogeneous three-dimensional distribution of complexes in the sample as expected for soluble proteins. Automated comparative analysis computes distance distributions by neural network analysis with DeerNet^105^ and Tikhonov regularization^106^ with DeerLab in a single step with background correction^107^ and provides 95% confidence intervals for both distributions. Unless otherwise indicated, we report the mean of the two distributions and confidence intervals that include uncertainty due to model bias. The mean distances (〈r〉) and standard deviations (σ(r)) of the distance distributions were implemented as upper and lower limits in CYANA modeling (see below).

### Small-angle scattering experiments

Small-angle neutron scattering experiments were recorded at the SANS-I and SANS-II facility, Swiss Spallation Neutron Source, SINQ, Paul Scherrer Institute, Switzerland. Scattering density matching points for RNA and protein with respect to the D_2_O/H_2_O ratio of the buffer (10 mM NaPO_4_, pH 6.5, 20 mM NaCl, 2 mM DTT) were determined by contrast variation and extrapolation of I_0_ to be 66% D_2_O for RNA, and 42% or 112% for protonated or deuterated protein, respectively. Free protein and RNA were recorded at a wavelength of the neutron beam of 6 Å, a collimation of 6 m and a detector distance of 2 m and 6 m. SAS data collection parameters are summarized in Supplementary Table 8.

For both, SANS and SAXS experiments, complexes were reconstituted as described previously^39^ and dialyzed for 24 h against suitable buffers. Each reference cuvette was filled with the corresponding dialysis buffer. Complexes were measured at SANS-I at a wavelength of 4.5 Å and either a collimation of 11 m and a detector distance of 11 m or a collimation of 3 m and a detector distance of 2 m. Scattered neutrons were detected using a two-dimensional 96 × 96 cm^2^ detector with a pixel size of 0.75 cm. The cross section of the collimator was 50 × 50 cm^2^ and the effective sample diameter was 0.8 × 1.5 cm^2^. At SANS-II, the selected wavelength was 4.9 Å and collimation and detector distance were 2 m and 1.2 m or 4 m and 4 m, respectively. The detector had a diameter of 64 cm with 128 × 128 pixels.

Reduction and analysis of SANS data were performed with the program BerSans^108^ and visualized using Primus QT^109^ from the ATSAS program^110^. Beamline-specific correction factors for data reduction are 1.338 and 1.284 for data recorded at a wavelength of 4.5 and 6 Å, respectively. SAXS experiments were performed on the liquid samples using a Rigaku MicroMax-002 microfocused beam (4 kW, 45 kV, 0.88 mA). The Cu Kα radiation (λ_CuKα_ = 1.5418 Å) was collimated by three pinholes (0.4, 0.3, and 0.8 mm) collimators. The scattered X-ray intensity was detected by a two-dimensional Triton-200 X-ray detector (20 cm diameter, 200 mm resolution) for SAXS. SAXS detectors cover an effective scattering vector range of 0.1 nm^−1^ < q < 4 nm^−1^, where q is the scattering wave vector defined as q = 4π sin θ/λ_CuKα_, with a scattering angle of 2θ. The SAXS samples were measured in 2 mm quartz X-ray capillaries (purchased from Hilgenberg).

Analysis of experimental data (R_g_, D_max_, Porod Volumes) was done using the ATSAS Suite, in particular Primus and functions therein^111^.

### Ensemble modeling with EOM

The SANS curve measured for protonated PTBP1 at 0% D_2_O was used for ensemble modeling with EOM. For this, the first models of each NMR structure of the free PTBP1-domains RRM1, RRM2 and RRM34 (pdb codes: 1SJQ, 1SJR and 2EVZ, respectively)^10,35^ were renumbered according to the PTBP1 amino acid sequence and used as rigid bodies, RRM34 (amino acids 324–531) was set as “fixed”. Sequence ranges 56–141, 177–284 and 324–531 were defined as rigid bodies, all other as “disordered”.

### Modeling with CYANA

For the integrative structure modeling of PTBP1/EMCV-IRES-DtoF (Supplementary Fig. 3), we used the previously determined CLIR-MS/MS derived models of PTBP1 RRM1, RRM3 and RRM4, as well as, the NMR-derived solution structure of RRM2 in complex with the respective RNA sites as rigid bodies^39^. However, as CYANA does not allow to load protein-RNA complexes as input structures, protein and RNA were loaded separately. Based on the imino-^1^H^1^H-NOESY signals and structure prediction by McFold/McSym, we modeled RNA stems using standard A-form RNA-helix angles and Watson-Crick base-pair restraints. We merged the protein models (only the first state of the bundle) and RNA models in one PDB file (numbering was adjusted to fit all models on a single chain as required for CYANA) and used the regularized macro of CYANA to generate a similar (but not identical) bundle of PTBP1 input structures (RMSD < 0.3 Å). Subsequently, the definition of rigid bodies in CYANA for protein domains was achieved using the command “angle fix”. To preserve the RNA secondary structure, Watson-Crick base-pair restraints were enforced in the stem, while RNA loops and bulges were kept flexible. These regularized rigid bodies were used as input for a CYANA calculation. The linkers between these rigid domains were generated with random starting conformations by CYANA. In these calculations, intermolecular restraints that were adapted from previous work^39^ were used to define protein-RNA contacts as observed in the individual sub-complexes.

To include EPR distance distributions in CYANA, we generated RRM models with 50 rotamers of the corresponding spin-labeled cysteine and calculated the geometric center of the radical positions. This average position was represented by a dummy glycine-Cα that was fixed by three or four strong restraints to the domain or linker-peptide, respectively (upper limits and lower limits in CYANA, ±0.05 Å of geometric center). A final set of 35 EPR distance restraints were implemented as upper and lower limits corresponding to the mean plus or minus one standard deviation of the distance distribution, respectively. Conformers are not penalized for their deviation from the mean distance as long as the distance is between the lower and upper limits.

As CYANA does not include electrostatics, we used artificial lower limits of 6 Å between RNA-phosphate groups with corresponding upper limits set to 200 Å. The latter distance is larger than D_max_ determined by SAS experiments and, thus, does not artificially compact the RNA.

Using these input rigid bodies (protein domains and RNA fragments) in combination with the short and long-range intermolecular restraints, we executed a simulated annealing protocol with 30,000 steps and calculated 20,000 models (20 × 1000 with different seeds). Out of these 20,000 models, the 10% energy best structures (lowest target function) were selected for further refinement. This filtering does not further bias the raw ensemble toward the EPR data.

### Structure refinement

All pseudoatoms from the CYANA output were removed, it was split into two chains (protein and RNA) and the RNA was renumbered by a customized script. Afterward, models with flexible peptide linkers that are threaded through RNA or contain C4’-O4’ bond lengths in RNA sugars longer than 1.6 Å were discarded. These tests discard between 2 and 8 models per run (out of 100 models). The models, which passed both tests, were refined by YASARA version 21.8.27 calling YASARA Structure with the standard script used by the YASARA Minimization Server and included in the MMMx distribution on GitHub as minimization_server.mcr. The resulting structure remains close to the input structure while removing clashes, and fixing wrong bond lengths, wrong bond angles, and wrong torsion angles.

The individual output models of YASARA have different protonation states of RNA, however, models in a PDB file must have the same number of atoms. Therefore, all protons that did not exist in all structures of the first run were removed to obtain consistent models with minimal protonation. Models with fewer protons than the first model, which usually indicates problems in RNA geometry, were discarded. Once again, models were checked for flexible peptide linkers threaded through RNA. This is necessary because some CYANA models were very strained, and their structures changed so strongly during refinement that such threading occurred even if it did not exist in the input model of the YASARA refinement. In the case of threading after YASARA refinement, models were discarded. Similarly, sugar bonds were checked again. When trying to remove strain, YASARA sometimes breaks a C4’-O4’ bond, because this is less costly in terms of energy than the alternatives. All models with altered sugar bonds were discarded. The set of all YASARA-refined CYANA models that passed these tests contained 766 conformers and furnished the raw ensemble for ensemble reweighting.

### Ensemble reweighting

Ensemble reweighting was performed with MMMx commit b330052. In total, 35 EPR (distance distribution) restraints, the SAXS curve and two SANS curves (66% D_2_O, 1.2 and 4 m detector distance) were used for refinement. An additional ensemble was refined only with the EPR restraints. In these refinements, the whole distance distribution, including its shape, is fitted, rather than only the mean and standard deviation.

Fitting was done in blocks of 100 conformers from the raw ensemble of 766 conformers with an algorithm searching for the global minimum by adjusting their populations^64^. In each such fit, all conformers with less than 1% of the population of the most populated conformer were discarded. Many populations were driven to exactly zero. Thus, after fitting one block of 100 conformers, a preliminary ensemble of much less than 100 conformers, together with corresponding populations, was obtained. This preliminary ensemble was topped up to obtain a new block of 100 conformers, by adding conformers from the raw ensemble that were not part of the first block of 100 conformers. The new block was fitted to obtain another preliminary ensemble with less than 100 conformers. This step was repeated multiple times, using up all conformers of the raw ensemble, with the last block usually containing less than 100 models.

In each block, the following fitting steps were performed. First, the block was fit only to the EPR restraints. This provides the best overlap deficiency^64^ with the conformers of this block by assigning populations (e.g., OD1). Overlap for a single distance distribution restraint corresponds to the common area below the experimental and simulated distance distributions, whereas the total area of each distribution is normalized to 1. It ranges between 0 for completely distinct and 1 for identical distributions. Overlap deficiency for the set of all distance distribution restraints ranges between 0 and 1, with 1 meaning no overlap for at least one restraint and 0 meaning perfect overlap for all restraints. Second, the block was fit to only the three small-angle scattering (SAS) curves by minimizing the sum of the *χ*^2^ values for all SAS curves. This provides the best sum of *χ*^2^ values that can be achieved for the conformers in this block by assigning populations (e.g., SCS1). Finally, the block was fit to EPR and SAS restraints simultaneously. In this fitting step, conformers of the block are selected, and populations are determined. Fit quality of EPR and SAS restraints are balanced. For each trial set of populations, the mean overlap deficiency (OD2) and the sum of *χ*^2^ values for the SAS curves (SCS2) are computed. Because this cannot be better than OD1 in this block, OD2 ≥ OD1. Likewise, it cannot be better than SCS1, i.e., SCS2 ≥ SCS1. Hence, (OD2/OD1 + SCS2/SCS1)/2 ≥ 1. The minimum of 1 can only occur if the same set of populations provides the best fit for EPR restraints as well as the best fit for SAS restraints. If the restraints are inconsistent or the set of input conformers is not good enough, the value will be substantially larger than 1. In order to get a transparent measure, we defined the loss of merit, (OD2/OD1 + SCS2/SCS1)/2 − 1. The loss of merit is zero, if both restraint sets are best fitted by the same set of conformers and their populations. If it is larger than one, the two figures of merit (overlap deficiency and sum of *χ*^2^ values) have more than doubled. If this happens, the subsets of restraints are inconsistent or the set of conformers is very poor. Values for the loss of merit of 0.2 to 0.4 are quite normal^112^. The loss of merit for the PTBP1/EMCV-IRES-DtoF ensemble refined with both DEER and SAS data is 0.332 and thus indicates overall consistent restraint sets and supports the integrity of the CYANA raw ensemble.

In a second approach to ensemble reweighting, we determined all conformer populations simultaneously by non-negative linear least squares (NNLLSQ). In this case, we fitted to small-angle scattering curves disregarding the dependence of noise on the scattering vector and to background-corrected DEER traces, which are output by automated comparative DEER analysis. The approach allows for an additional polynomial background correction, which we performed up to order 2. This approach was newly implemented into MMMx. It turned out that only a small fraction of the populations (48 out of 766) deviated significantly from zero (Supplementary Fig. 13). The NNLLSQ approach balances the loss of fit quality between individual data sets, whereas the non-linear approach balances it between groups of similar restraints (distance distributions and small-angle scattering curves). Since we have 35 distance distributions, but only 3 scattering curves, NNLLSQ favors overlap with the distance distributions at the expense of increased *χ*^2^ of the small-angle scattering curve fits. In this work, we prefer the non-linear approach that balances between groups of restraints for the ensembles specified as the main ensemble. MMMx is freely available at https://github.com/gjeschke/MMMx.

### Ensemble comparison and assessment of ensemble quality

Radii of gyration for all ensembles (Supplementary Table 9) and overlap of pseudo-electron density for all pairs of ensembles were computed with the EnsembleAnalysis module of MMMx commit 330052. MolProbity was downloaded from GitHub (https://github.com/rlabduke/MolProbity, accessed Dec 21, 2022). The command line tool online analysis was used to provide quality measures for the individual conformers. Quality measures for the ensemble, as reported in Supplementary Table 10, were obtained by population-weighted averaging over the ensemble with the newly introduced MMMx function rd_MolProbity_results.m. MMMx is freely available at https://github.com/gjeschke/MMMx.

### Reporting summary

Further information on research design is available in the Nature Portfolio Reporting Summary linked to this article.

### Supplementary information

Supplementary Information file

Reporting Summary

### Source data

Source Data

## Data Availability

As the integrative structure modeling approach is not listed as an accepted experimental method for structure generation by the wwPDB, the structural ensembles have been deposited in Zenodo (10.5281/zenodo.7798970). Further data supporting the findings of this study are available in the Supplementary Information. All the DEER, NMR, and SAS data are publicly available through the Zenodo repository (10.5281/zenodo.8379933)^113^. Additionally, the MMMx script for ensemble fitting was uploaded to Zenodo under the same DOI. The reanalyzed mass spectrometry proteomics data have been deposited to the ProteomeXchange Consortium (http://proteomecentral.proteomexchange.org) via the PRIDE partner repository with the dataset identifier PXD034894. PDB codes of previously published structures used in this study are 1SJQ, 1SJR and 2EVZ. Other source data are provided with this paper as Source Data file. Source data are provided with this paper.

## Code availability

The MMMx suite used for structure refinement, ensemble reweighting, and ensemble comparison is available through the Zenodo at 10.5281/zenodo.8380079.
