# Supplementary material for: Integrative solution structure of PTBP1-IRES complex reveals strong compaction and ordering with residual conformational flexibility
Source: Nat Commun. 2023 Oct 13;14:6429. doi: 10.1038/s41467-023-42012-z (PMC10576089; doi:10.1038/s41467-023-42012-z)
Supplement: Supplementary file 1 — Supplementary Information file [file 41467_2023_42012_MOESM1_ESM.pdf]

Integrative solution structure of PTBP1-IRES complex reveals strong compaction and ordering with residual conformational flexibility

Georg Dorn[1,6], Christoph Gmeiner[2,6], Tebbe de Vries[1,6], Emil Dedic[1], Mihajlo Novakovic[1], Fred F. Damberger[1], Christophe Maris[1], Esteban Finol[1], Chris P. Sarnowski[3], Joachim Kohlbrecher[4], Timothy J. Welsh[2], Sreenath Bolisetty[5], Raffaele Mezzenga[5], Ruedi Aebersold[3], Alexander Leitner[3], Maxim Yulikov[2,*], Gunnar Jeschke[2,*], Frédéric H.-T. Allain[1,*]

[1]Institute of Biochemistry, Department of Biology, ETH Zürich, Zürich, Switzerland
[2]Laboratory of Physical Chemistry, Department of Chemistry and Applied Biosciences, ETH Zürich, Zürich, Switzerland
[3]Institute of Molecular Systems Biology, Department of Biology, ETH Zürich, Zürich, Switzerland
[4]Laboratory for Neutron Scattering and Imaging, Paul Scherrer Institut, Villigen, Switzerland
[5]Laboratory of Food & Soft Materials, Institute of Food, Nutrition and Health, Department for Health Sciences and Technology, ETH Zürich, Zürich, Switzerland
[6]These authors contributed equally
*corresponding authors

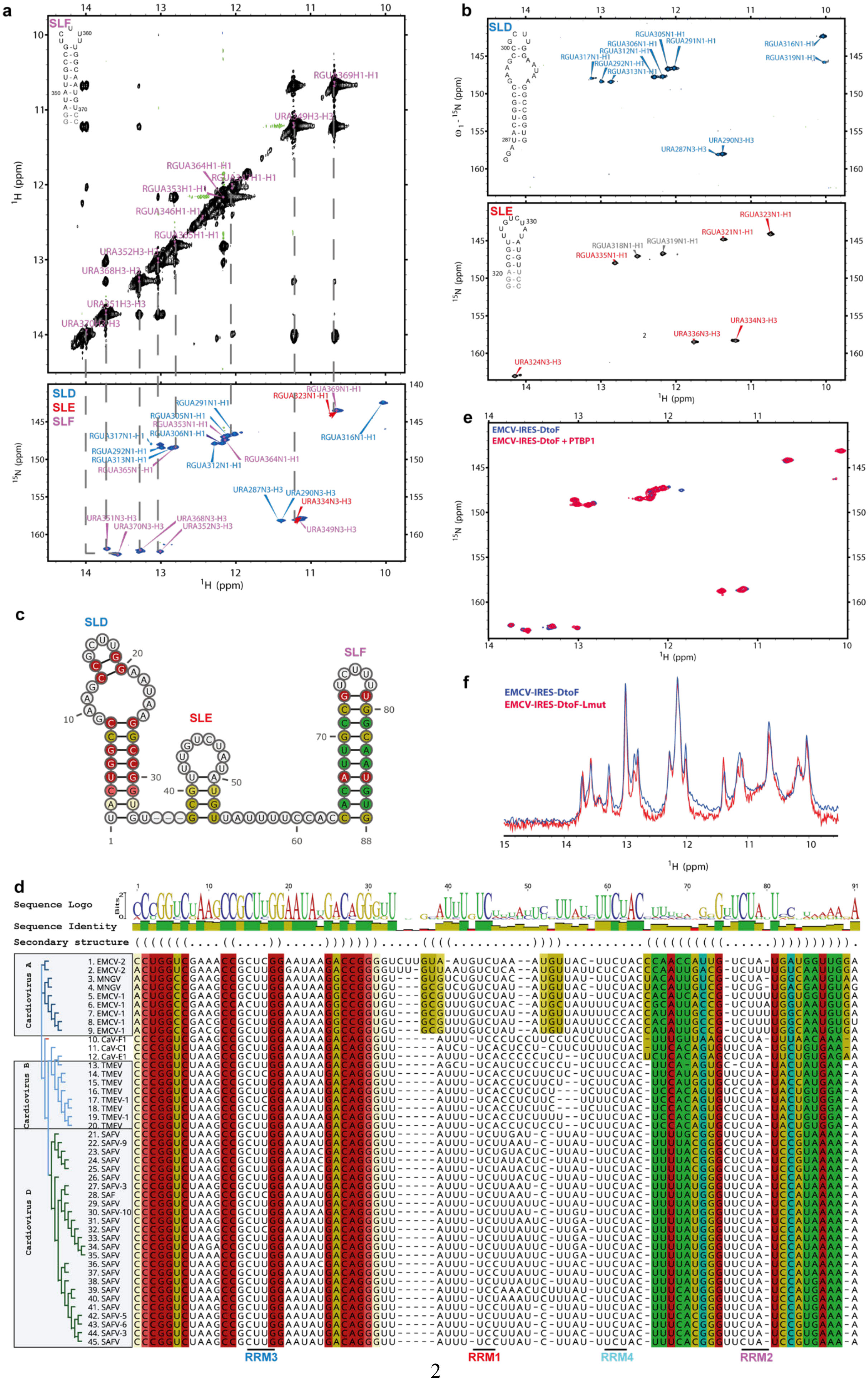
a
b
c
d
e
f
SLF
SLD
SLE
1H (ppm)
15N (ppm)
EMCV-IRES-DtoF
EMCV-IRES-DtoF + PTBP1
EMCV-IRES-DtoF-Lmut
Sequence Logo
Sequence Identity
Secondary structure
Cardiovirus A
Cardiovirus B
Cardiovirus D
RRM3
RRM1
RRM4
RRM2

Supplementary Fig. 1. Secondary structure probing and phylogeny of EMCV-IRES-DtoF. a Assignment of imino proton resonances using a 2D ^{1}H-^{1}H NOESY experiment (top) and standard "sequential walk" exemplified on SLF. Signals could be readily transferred to the 2D ^{1}H-^{15}N HSQC of the full-length RNA (bottom). **b** Transfer of imino proton assignments to the corresponding 2D ^{1}H-^{15}N HSQC of each SL served as validation and comparison with the typical imino resonance shifts of A-U, G-C and G-U base-pairs and enabled unambiguous assignment. The match of the imino proton resonances recorded on the individual stem loops as well as on the complete RNA sequence showed that the proposed secondary structures of the RNA are present in solution. **c** Consensus secondary structure for these RNA elements in the genome of cardioviruses A (EMCV-1, EMCV2 and MNGV). The base-pairs are color-coded on the predicted minimum free energy structure according to base-pair probabilities from the multiple sequence alignment (MSA), warm colours mean high probability and cold colours mean low probability. **d** MSA from unique sequences in the 5' UTR of all cardioviruses. Sequence logo, sequence identity (conservation) and secondary structure in dot-bracket annotation are shown on top. Columns of aligned RNA bases are colour-coded to correspond with the consensus RNA secondary structure. Complete genome sequences were retrieved from NCBI database using the key word "Cardiovirus". An MSA was built using MAFFT software and it was trimmed to include the SLD, E and F in the 5' UTR of the cardioviruses genome only. RNAalifold software100 was used to obtain the consensus secondary structure and its predicted free energy. Sequence logo and sequence identity were computed using geneious 2022.1.1 software. Sequences used for the alignment are listed in Supplementary Table 1. The binding sites of the RRMs of PTBP1 according to39 are indicated below the alignment. **e** 2D-^{1}H-^{15}N-TROSY spectra of free EMCV-IRES-DtoF and bound to PTBP1. Imino ^{1}H-^{15}N signals of the free EMCV-RNA (blue) and the PTBP1-EMCV-complex (red) remain at the same chemical shift in proton and nitrogen. This strongly indicates that the local RNA stem-loop structures remain unchanged upon protein binding. The NMR spectra were recorded at 288 K. The cross-peak at 11.7 ppm/147 ppm present in the free RNA originate for an imino associated with 3'end heterogeneity of the RNA. This peak is absent in the complex probably due to exchange broadening. **f** Overlay of 1D-^{1}H-spectra recorded on EMCV-RNA with pyrimidine-to-purine mutations in LinkEF (Lmut, red) and wild-type (WT, blue) RNA. The signature of the imino-protons remains the same, demonstrating that the secondary structure elements of the RNA were not affected by the mutations of the single stranded LinkEF region.

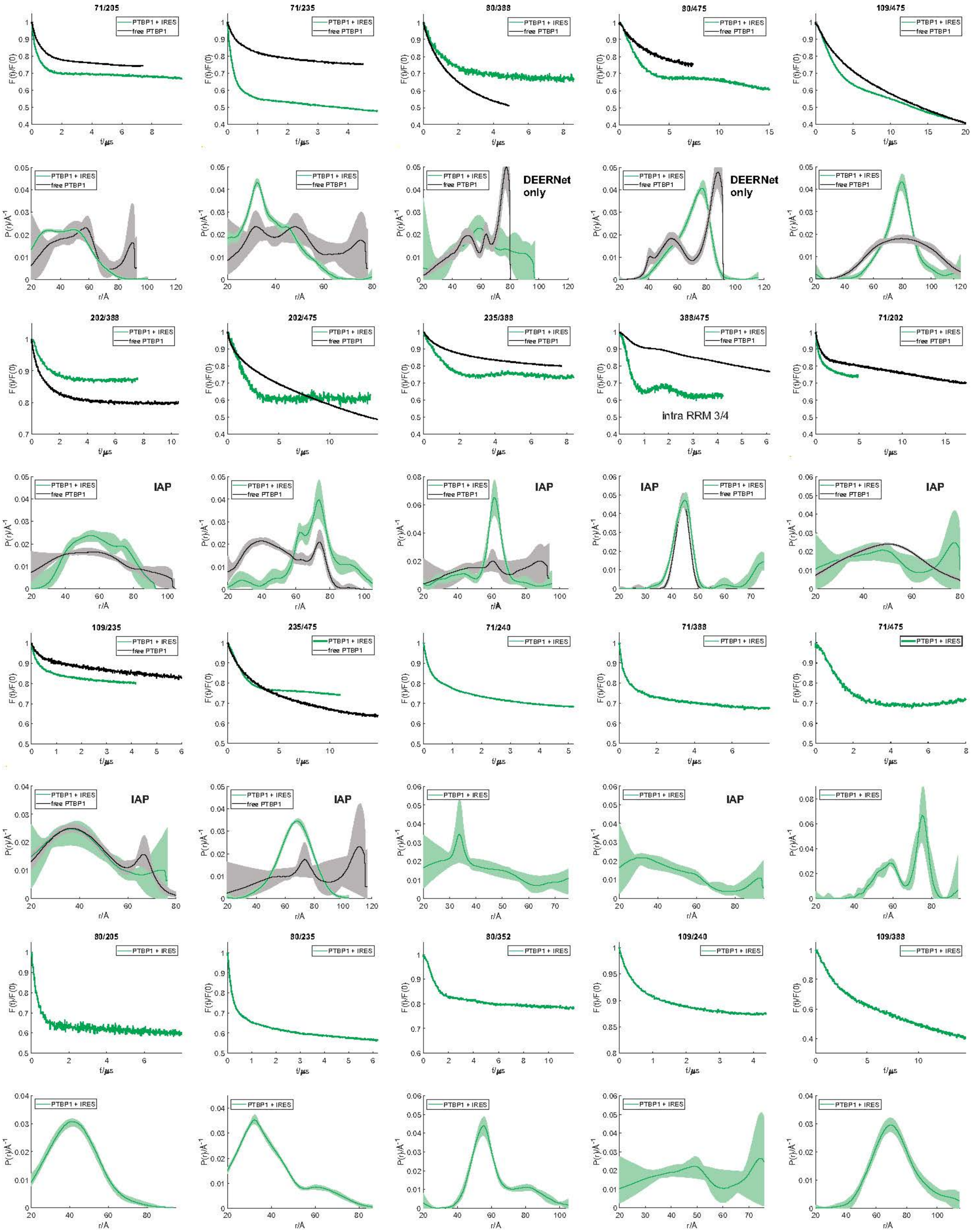

71/205
71/235
80/388
80/475
109/475
202/388
202/475
235/388
388/475
71/202
109/235
235/475
71/240
71/388
71/475
80/205
80/235
80/352
109/240
109/388
PTBP1 + IRES
free PTBP1
DEERNet only
IAP
intra RRM 3/4
F(t)/F(0)
t/μs
P(r)/Å-1
r/Å

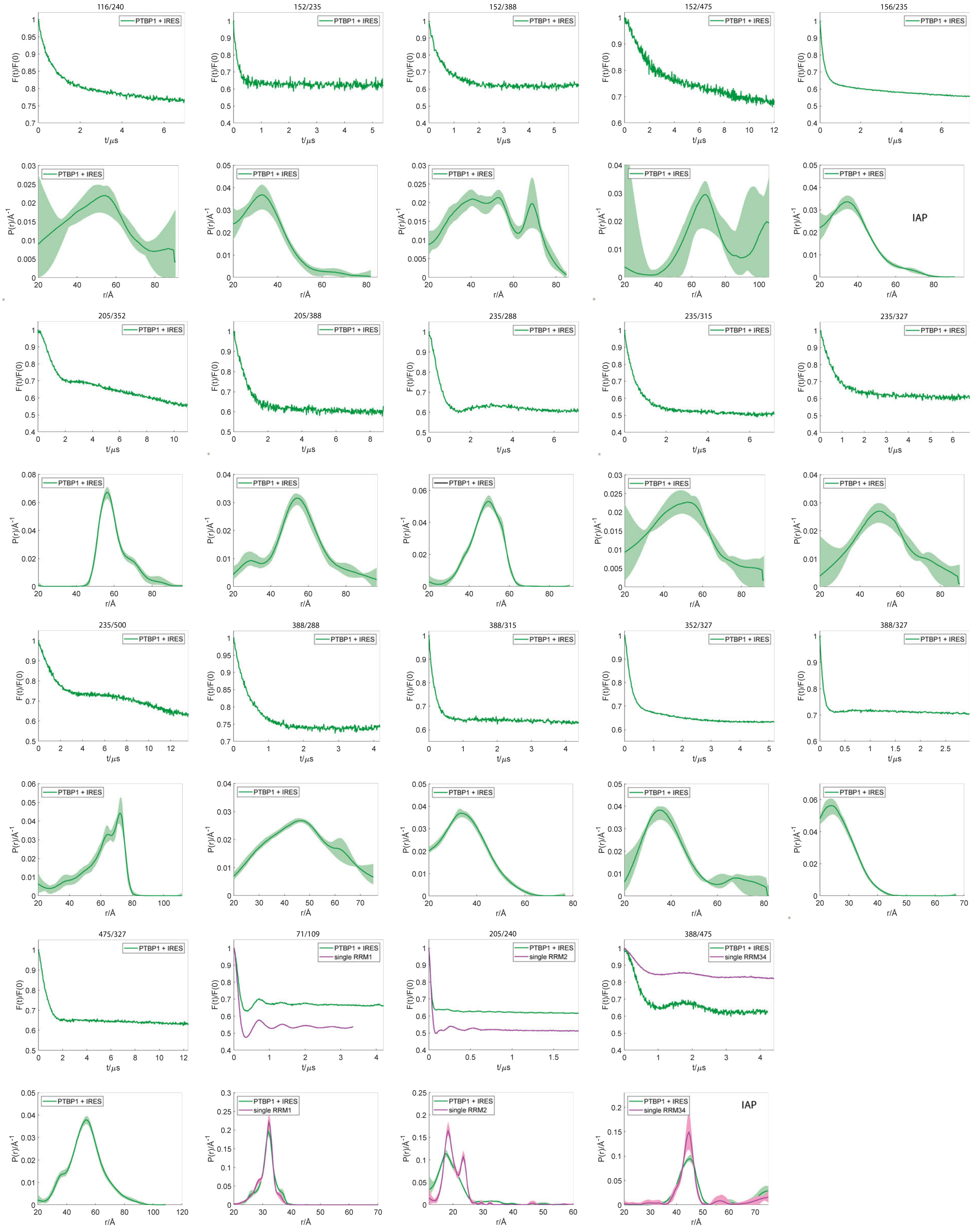

Supplementary Fig. 2. Distance distributions calculated from DEER-EPR data recorded on free PTBP1 and the PTBP1-EMCV complex. Primary time-domain data are shown above the distributions. Green color denotes the complex and black color the free protein. Semi-transparent areas correspond to 95% confidence intervals. Samples with iodoacetamido proxyl spin label are indicated by “IAP”. Distance distributions computed by only neural network analysis with DeerNet are indicated.

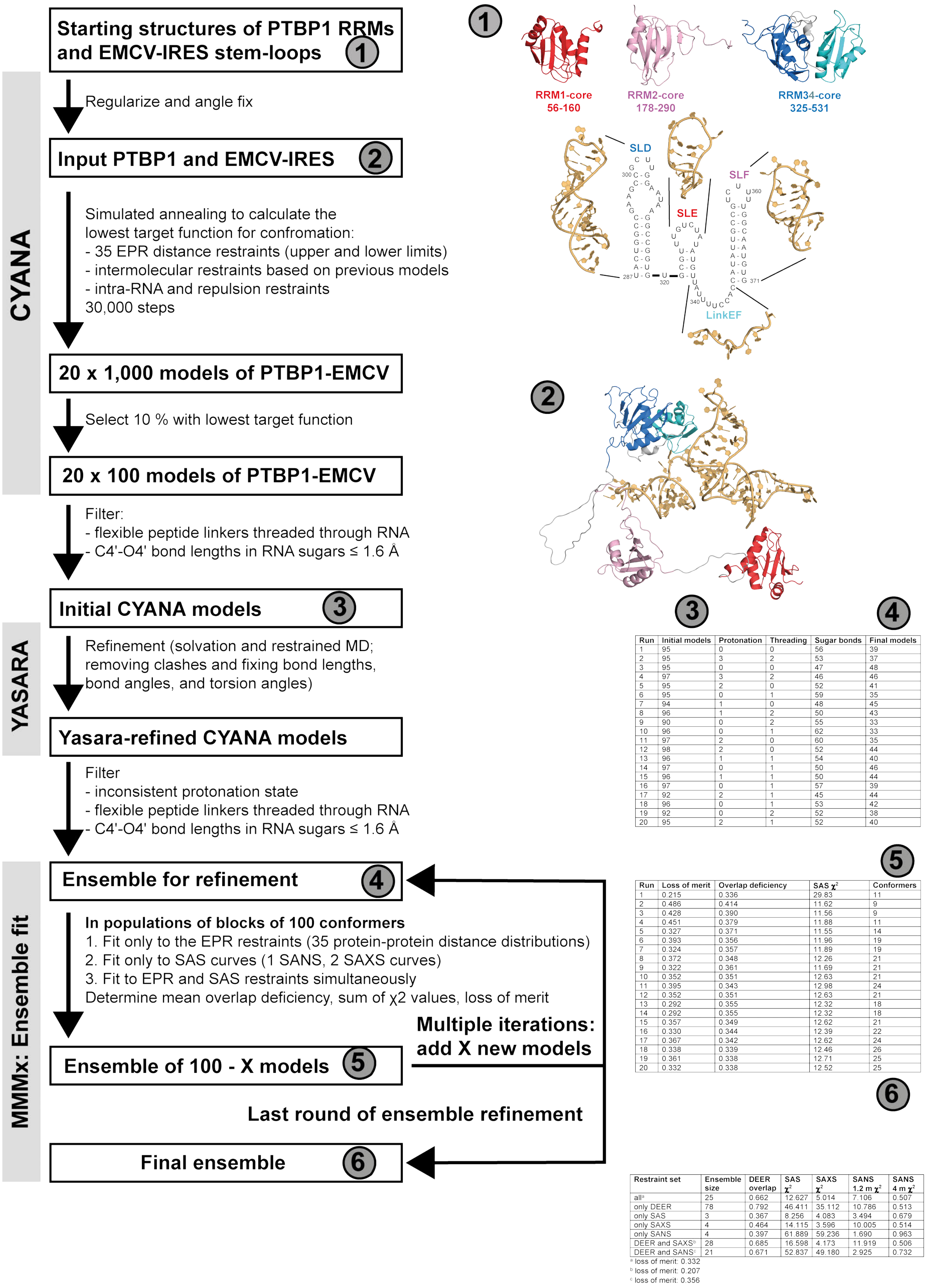

Run	Initial models	Protonation	Threading	Sugar bonds	Final models
1	95	0	0	56	39
2	95	3	2	53	37
3	95	0	0	47	48
4	97	3	2	46	46
5	95	2	0	52	41
6	95	0	1	59	35
7	94	1	0	48	45
8	96	1	2	50	43
9	90	0	2	55	33
10	96	0	1	62	33
11	97	2	0	60	35
12	98	2	0	52	44
13	96	1	1	54	40
14	97	0	1	50	46
15	96	1	1	50	44
16	97	0	1	57	39
17	92	2	1	45	44
18	96	0	1	53	42
19	92	0	2	52	38
20	95	2	1	52	40

Run	Loss of merit	Overlap deficiency	SAS χ^2	Conformers
1	0.215	0.336	29.83	11
2	0.486	0.414	11.62	9
3	0.428	0.390	11.56	9
4	0.451	0.379	11.88	11
5	0.327	0.371	11.55	14
6	0.393	0.356	11.96	19
7	0.324	0.357	11.89	19
8	0.372	0.348	12.26	21
9	0.322	0.361	11.69	21
10	0.352	0.351	12.63	21
11	0.395	0.343	12.98	24
12	0.352	0.351	12.63	21
13	0.292	0.355	12.32	18
14	0.292	0.355	12.32	18
15	0.357	0.349	12.62	21
16	0.330	0.344	12.39	22
17	0.367	0.342	12.62	24
18	0.338	0.339	12.46	26
19	0.361	0.338	12.71	25
20	0.332	0.338	12.52	25

Restraint set	Ensemble size	DEER overlap	SAS χ^2	SAXS χ^2	SANS 1.2 m χ^2	SANS 4 m χ^2
all[a]	25	0.662	12.627	5.014	7.106	0.507
only DEER	78	0.792	46.411	35.112	10.786	0.513
only SAS	3	0.367	8.256	4.083	3.494	0.679
only SAXS	4	0.464	14.115	3.596	10.005	0.514
only SANS	4	0.397	61.889	59.236	1.690	0.963
DEER and SAXS[b]	28	0.685	16.598	4.173	11.919	0.506
DEER and SANS[c]	21	0.671	52.837	49.180	2.925	0.732

[a] loss of merit: 0.332
[b] loss of merit: 0.207
[c] loss of merit: 0.356

Supplementary Fig. 3. Scheme of the integrative structural modeling approach. For details see Materials and Methods. For ensemble fitting, the number of iterations depends on the number of models in the preliminary ensembles and cannot be predicted.

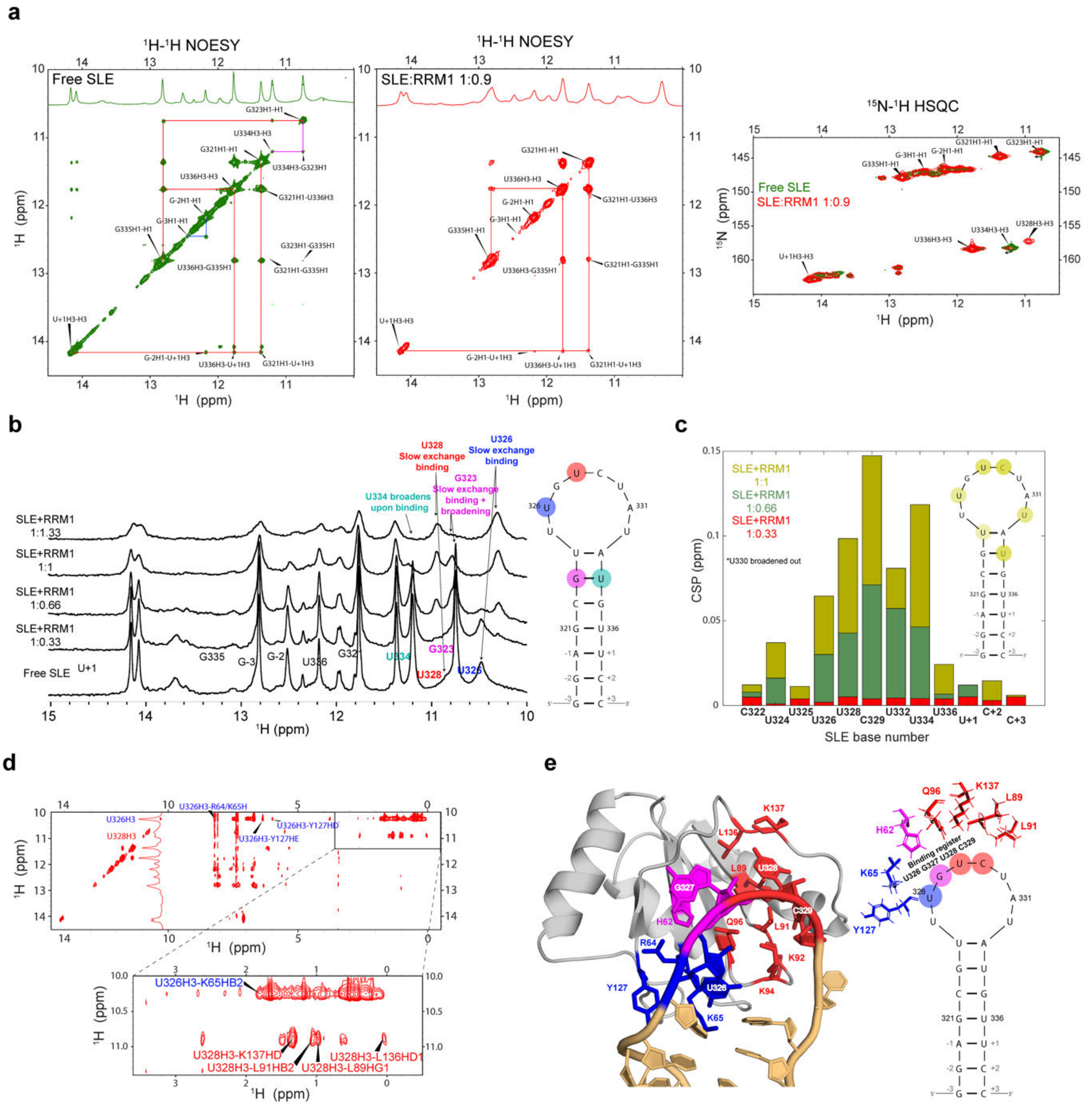

Supplementary Fig. 4. NMR confirmation of the PTBP1 RRM1/SLE binding register. a The secondary structure of SLE stem remains unaffected upon binding with RRM1 as shown by 2D ^{1}H-^{1}H NOESY imino proton sequential walk and 2D ^{1}H-^{15} NHSQC spectra at 278 K. Even though upon binding, the G323 and U334 wobble base pair do not give a characteristic NOE cross-peak in 2D ^{1}H-^{1}H NOESY spectra (probably due to signal broadening caused by increased correlation time upon binding), these resonances are still present in a 2D ^{1}H-^{15}N HSQC spectrum. This indicates that they are still forming a base pair and consequently implies that U324 and A333 also remain base paired despite the absence of an assigned U324-H3 signal. Correlations labeled with asterisk are assigned using a recently developed SMT imino experiment. **b** Titration of SLE with RRM1. Perturbations of SLE imino proton signals upon binding at 278 K led to important conclusions about the binding interface. U326 and U328 are in the slow exchange binding regime and remain sharp due to direct stabilizing interaction with protein. Imino signals of U326 and U328 are assigned according to intermolecular NOE signals with RRM1. G323 and U334 imino proton signals experience additional broadening due to slightly faster chemical exchange of respective imino hydrogens with water. **c** CSP perturbations of H5-H6 correlations. Plot of H5-H6 chemical shift perturbations upon addition of RRM1 calculated from correlations in the ^{1}H-^{1}H TOCSY experiment. Strongest perturbations are observed for U326, U328, C329, U332 and U334 implying largest conformational changes involving mostly bases within the apical loop upon protein-RNA interaction. **d** Intermolecular L-PROSY NOESY, identifying U326 and U328 imino proton signals**.** Intermolecular NOE contacts involving imino proton resonances acquired using L-PROSY NOESY experiment at 278 K. Cross-peaks detected for the protein can be used to facilitate assignment of two imino proton resonances from the apical loop, U326 and U328. **e** Mapping of

binding interface based on NOESY correlations. Multiple intermolecular NOESY experiments acquired in H_2O and D_2O (at 278 K and 323 K, respectively) reveal intermolecular correlations between protein and RNA. These intermolecular contacts are highlighted on the 3D structural model. All NMR data support the binding register that is illustrated on the secondary structure of SLE.

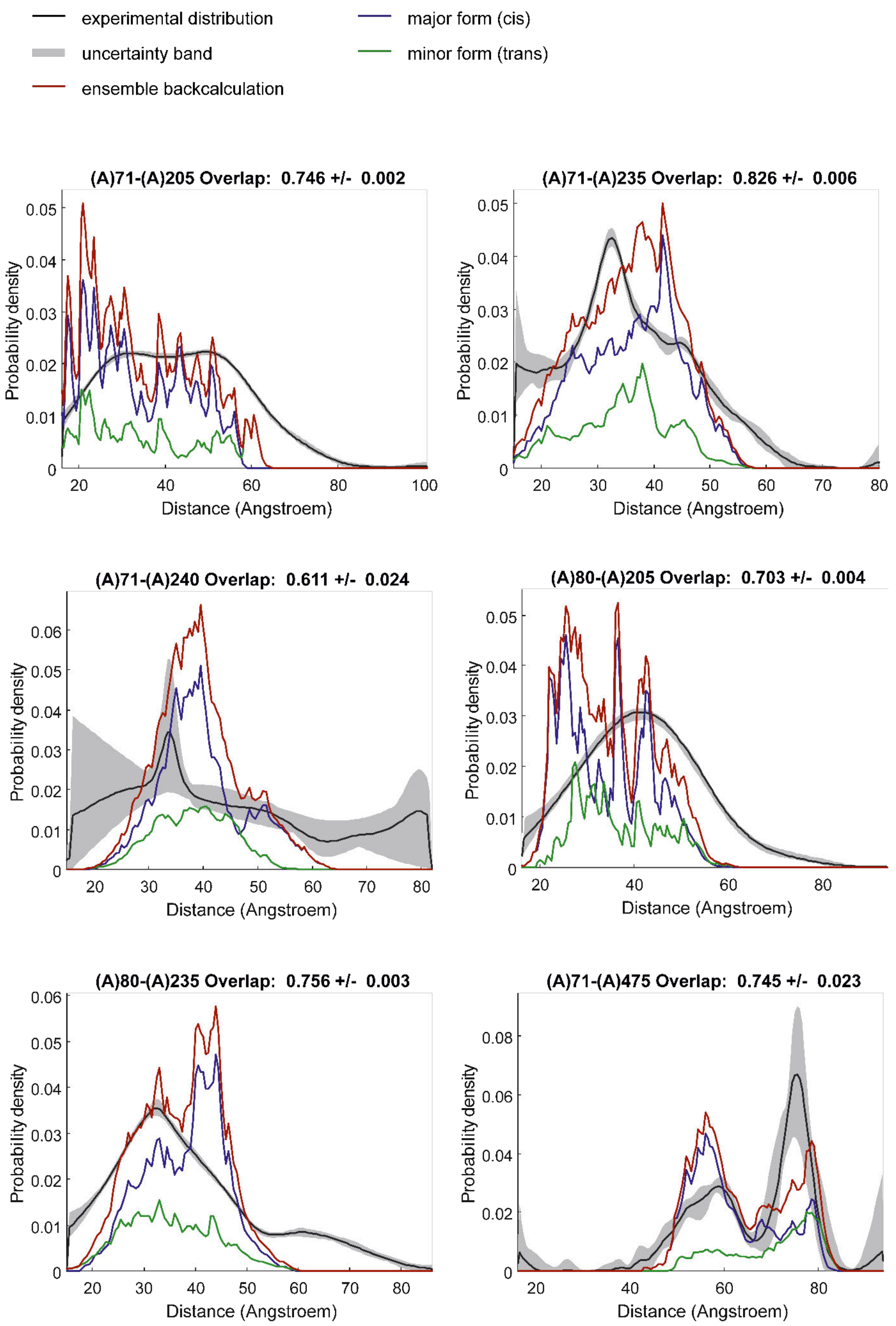
experimental distribution
uncertainty band
ensemble backcalculation
major form (cis)
minor form (trans)
(A)71-(A)205 Overlap: 0.746 +/- 0.002
(A)71-(A)235 Overlap: 0.826 +/- 0.006
(A)71-(A)240 Overlap: 0.611 +/- 0.024
(A)80-(A)205 Overlap: 0.703 +/- 0.004
(A)80-(A)235 Overlap: 0.756 +/- 0.003
(A)71-(A)475 Overlap: 0.745 +/- 0.023
Probability density
Distance (Angstroem)

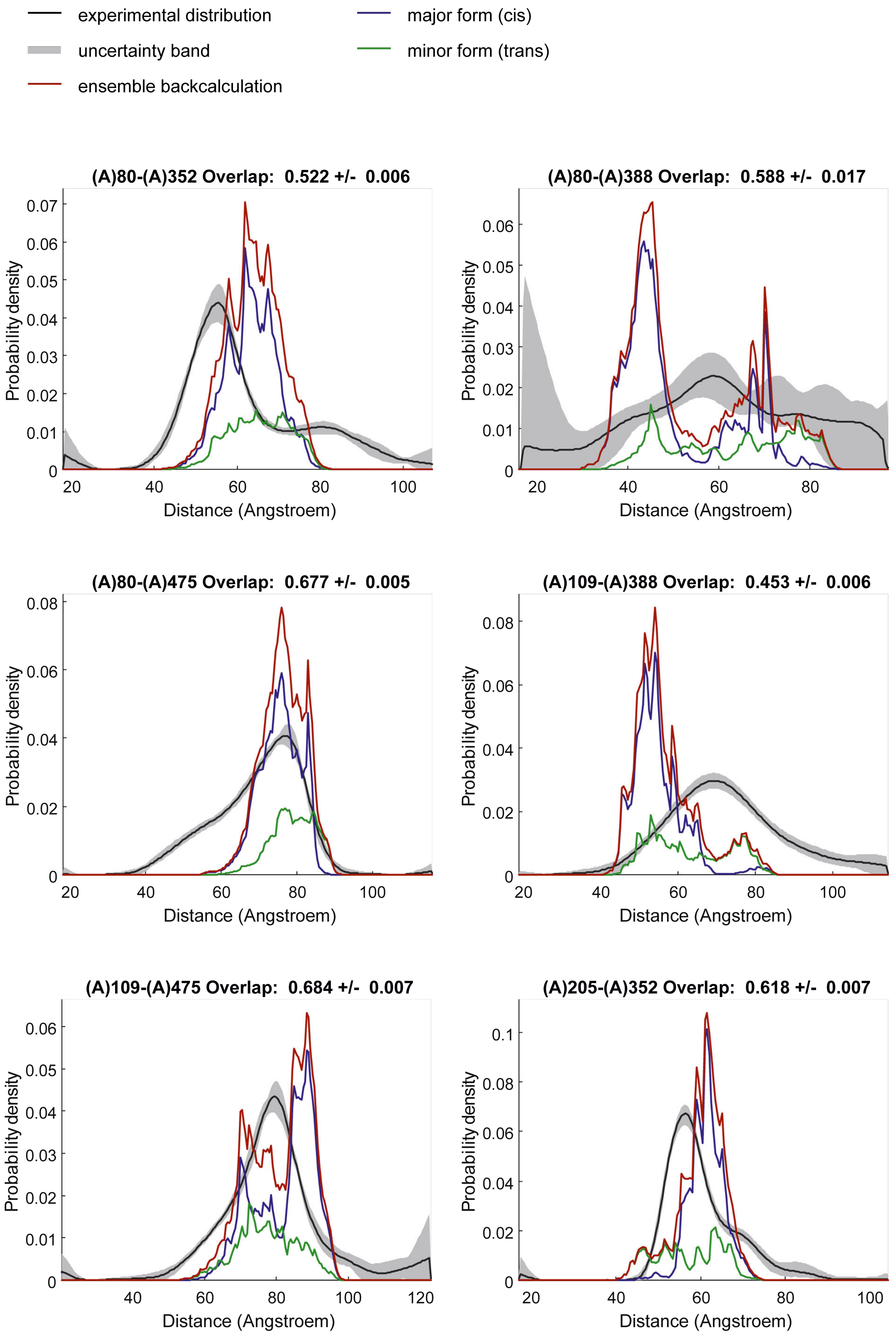

experimental distribution
uncertainty band
ensemble backcalculation
major form (cis)
minor form (trans)
(A)80-(A)352 Overlap: 0.522 +/- 0.006
(A)80-(A)388 Overlap: 0.588 +/- 0.017
(A)80-(A)475 Overlap: 0.677 +/- 0.005
(A)109-(A)388 Overlap: 0.453 +/- 0.006
(A)109-(A)475 Overlap: 0.684 +/- 0.007
(A)205-(A)352 Overlap: 0.618 +/- 0.007
Probability density
Distance (Angstroem)

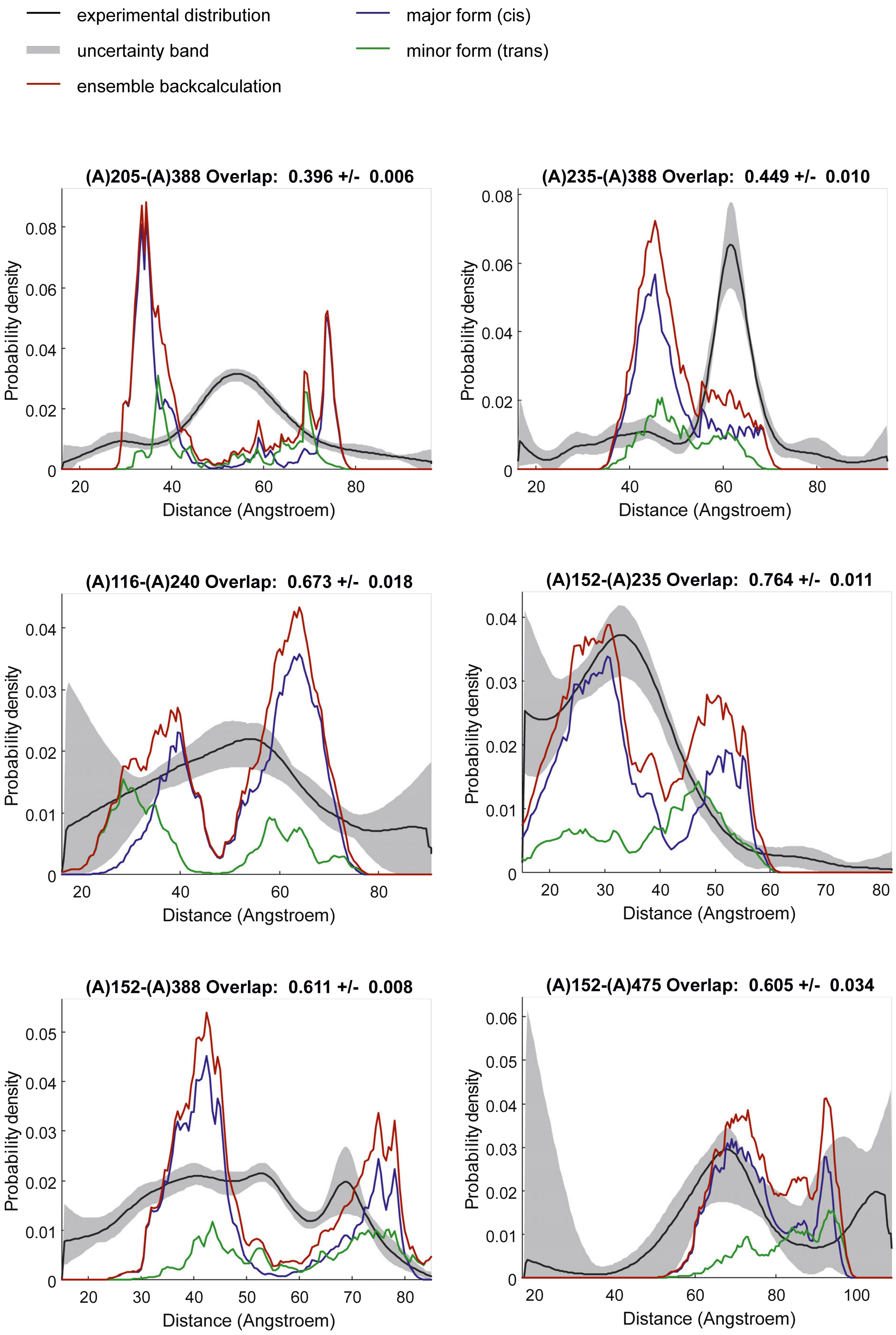
experimental distribution
uncertainty band
ensemble backcalculation
major form (cis)
minor form (trans)
(A)205-(A)388 Overlap: 0.396 +/- 0.006
(A)235-(A)388 Overlap: 0.449 +/- 0.010
(A)116-(A)240 Overlap: 0.673 +/- 0.018
(A)152-(A)235 Overlap: 0.764 +/- 0.011
(A)152-(A)388 Overlap: 0.611 +/- 0.008
(A)152-(A)475 Overlap: 0.605 +/- 0.034
Probability density
Distance (Angstroem)

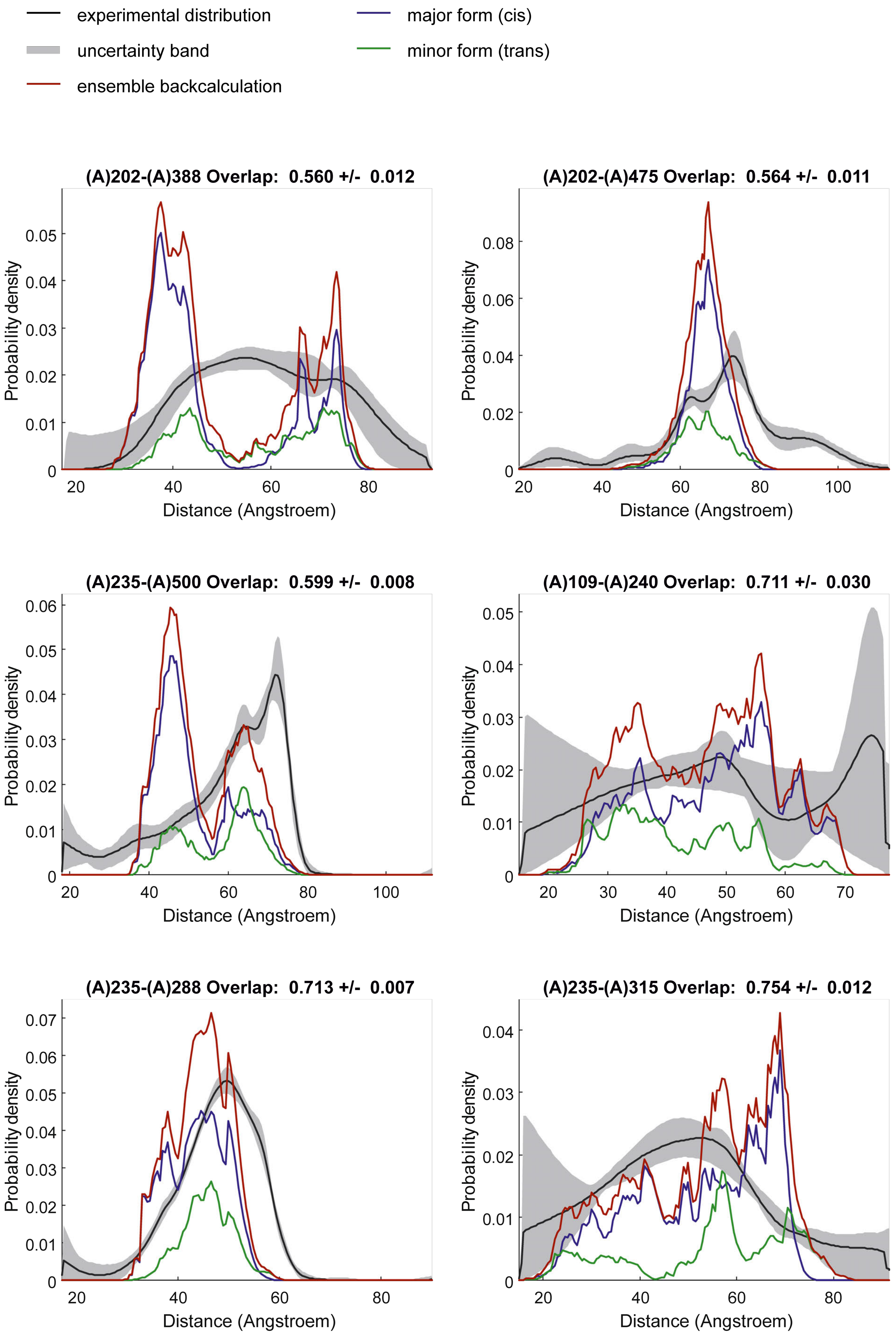
experimental distribution
uncertainty band
ensemble backcalculation
major form (cis)
minor form (trans)
(A)202-(A)388 Overlap: 0.560 +/- 0.012
(A)202-(A)475 Overlap: 0.564 +/- 0.011
(A)235-(A)500 Overlap: 0.599 +/- 0.008
(A)109-(A)240 Overlap: 0.711 +/- 0.030
(A)235-(A)288 Overlap: 0.713 +/- 0.007
(A)235-(A)315 Overlap: 0.754 +/- 0.012
Probability density
Distance (Angstroem)

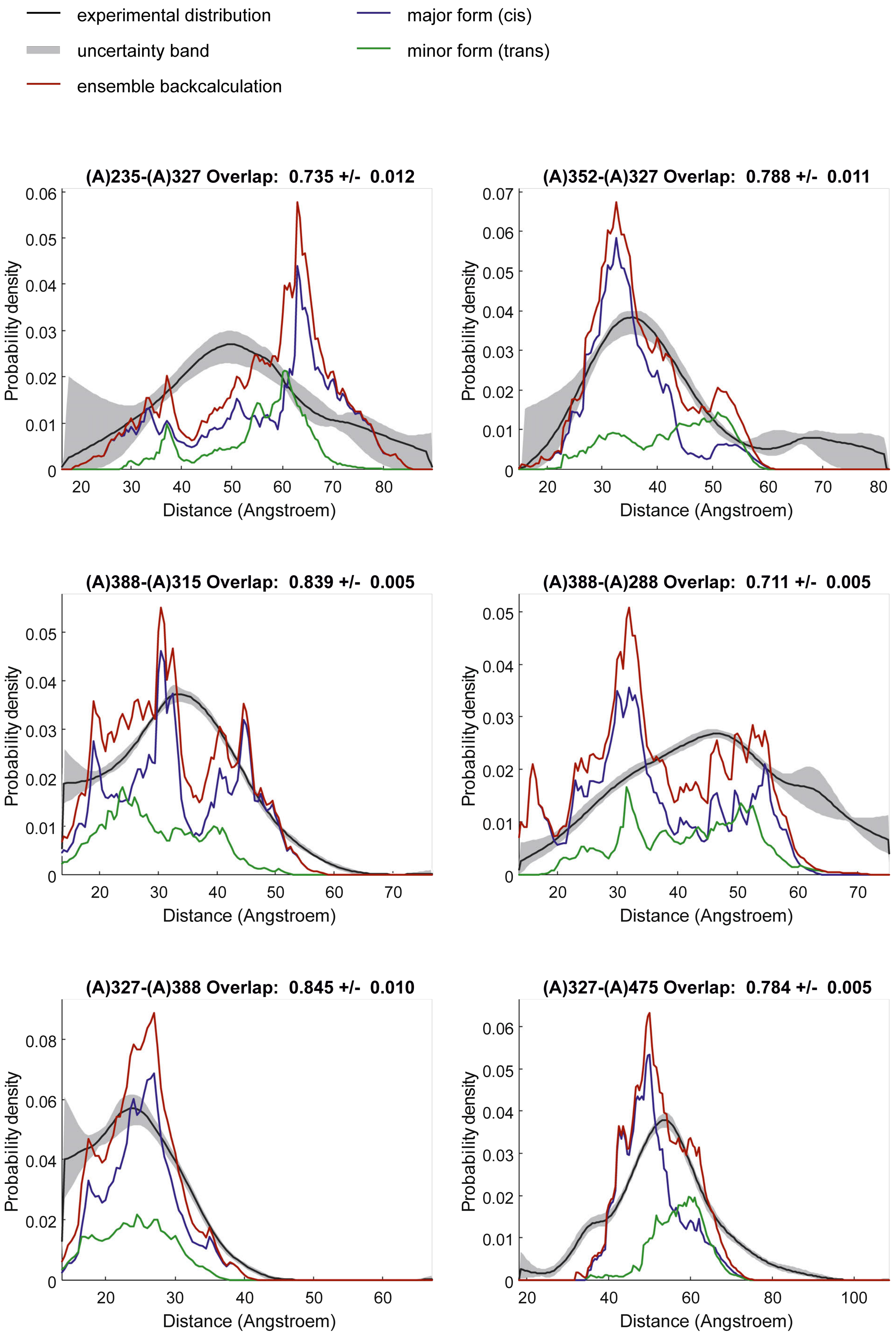

experimental distribution
uncertainty band
ensemble backcalculation
major form (cis)
minor form (trans)
(A)235-(A)327 Overlap: 0.735 +/- 0.012
(A)352-(A)327 Overlap: 0.788 +/- 0.011
(A)388-(A)315 Overlap: 0.839 +/- 0.005
(A)388-(A)288 Overlap: 0.711 +/- 0.005
(A)327-(A)388 Overlap: 0.845 +/- 0.010
(A)327-(A)475 Overlap: 0.784 +/- 0.005
Probability density
Distance (Angstroem)

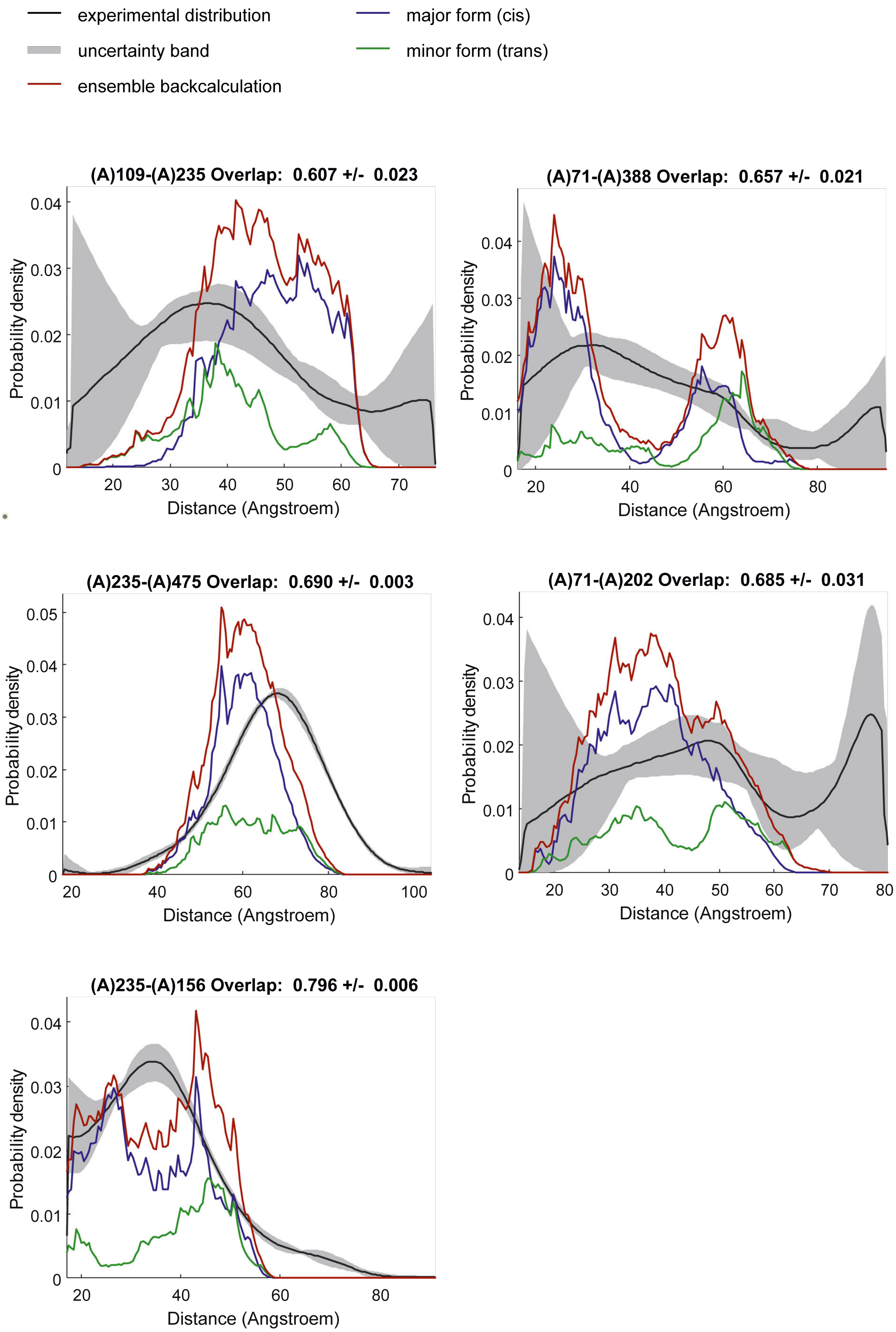
experimental distribution
uncertainty band
ensemble backcalculation
major form (cis)
minor form (trans)
(A)109-(A)235 Overlap: 0.607 +/- 0.023
(A)71-(A)388 Overlap: 0.657 +/- 0.021
(A)235-(A)475 Overlap: 0.690 +/- 0.003
(A)71-(A)202 Overlap: 0.685 +/- 0.031
(A)235-(A)156 Overlap: 0.796 +/- 0.006
Probability density
Distance (Angstroem)

Supplementary Fig. 5. Restraint fit of the integrative PTBP1/EMCV-IRES-DtoF ensemble with DEER distance distributions. Experimentally determined distance distribution (black line) with corresponding uncertainty (grey shading) and predicted for the entire ensemble (red line) or only cis (group 1, purple) and trans (group 2, green) sub-groups, respectively. Overlap values between experimental and predicted distributions for each spin pair are displayed at the top of each panel together with their uncertainty arising from only the uncertainty of the experimental distribution.

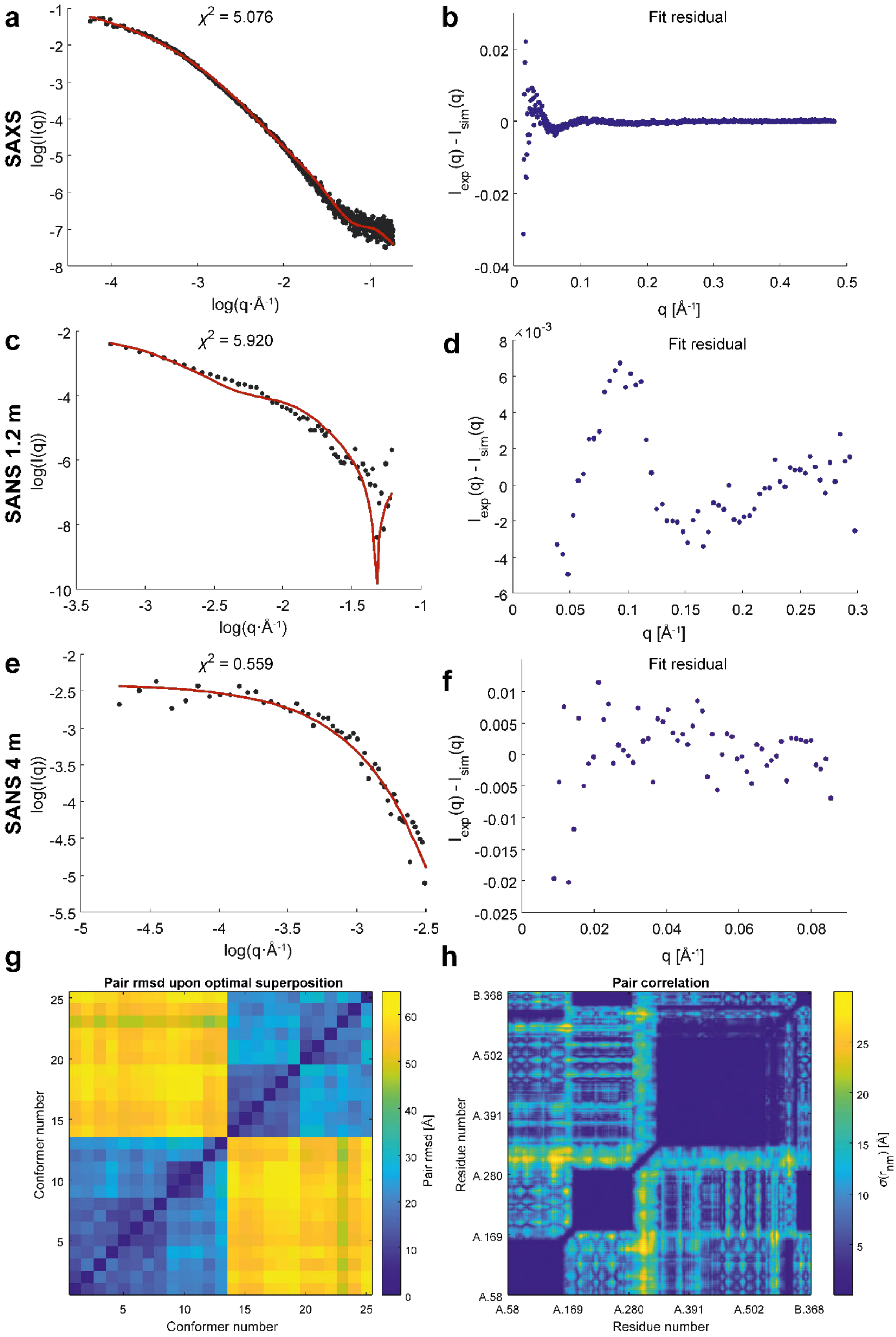
a
SAXS
log(I(q))
log(q·Å-1)
χ2 = 5.076
b
Fit residual
Iexp(q) - Isim(q)
q [Å-1]
c
SANS 1.2 m
log(I(q))
log(q·Å-1)
χ2 = 5.920
d
×10-3
Fit residual
Iexp(q) - Isim(q)
q [Å-1]
e
SANS 4 m
log(I(q))
log(q·Å-1)
χ2 = 0.559
f
Fit residual
Iexp(q) - Isim(q)
q [Å-1]
g
Pair rmsd upon optimal superposition
Conformer number
Conformer number
Pair rmsd [Å]
h
Pair correlation
Residue number
Residue number
A.58
A.169
A.280
A.391
A.502
B.368
σ(rnm) [Å]

Supplementary Fig. 6. Restraint fit and residuals of the integrative PTBP1/EMCV-IRES-DtoF ensemble with SANS/SAXS data as well as pair-wise correlation. Fit **a** and residual **b** of ensemble with SAXS, SANS 1.2 m (**c**,**d**), and SANS 4 m (**e**,**f**). Red curves were back-calculated from the ensemble. **g** Conformer pair root mean square deviation (Å). **h** Pair correlation matrix of the ensemble. Distances (Å) were measured between Cα of the two residues.

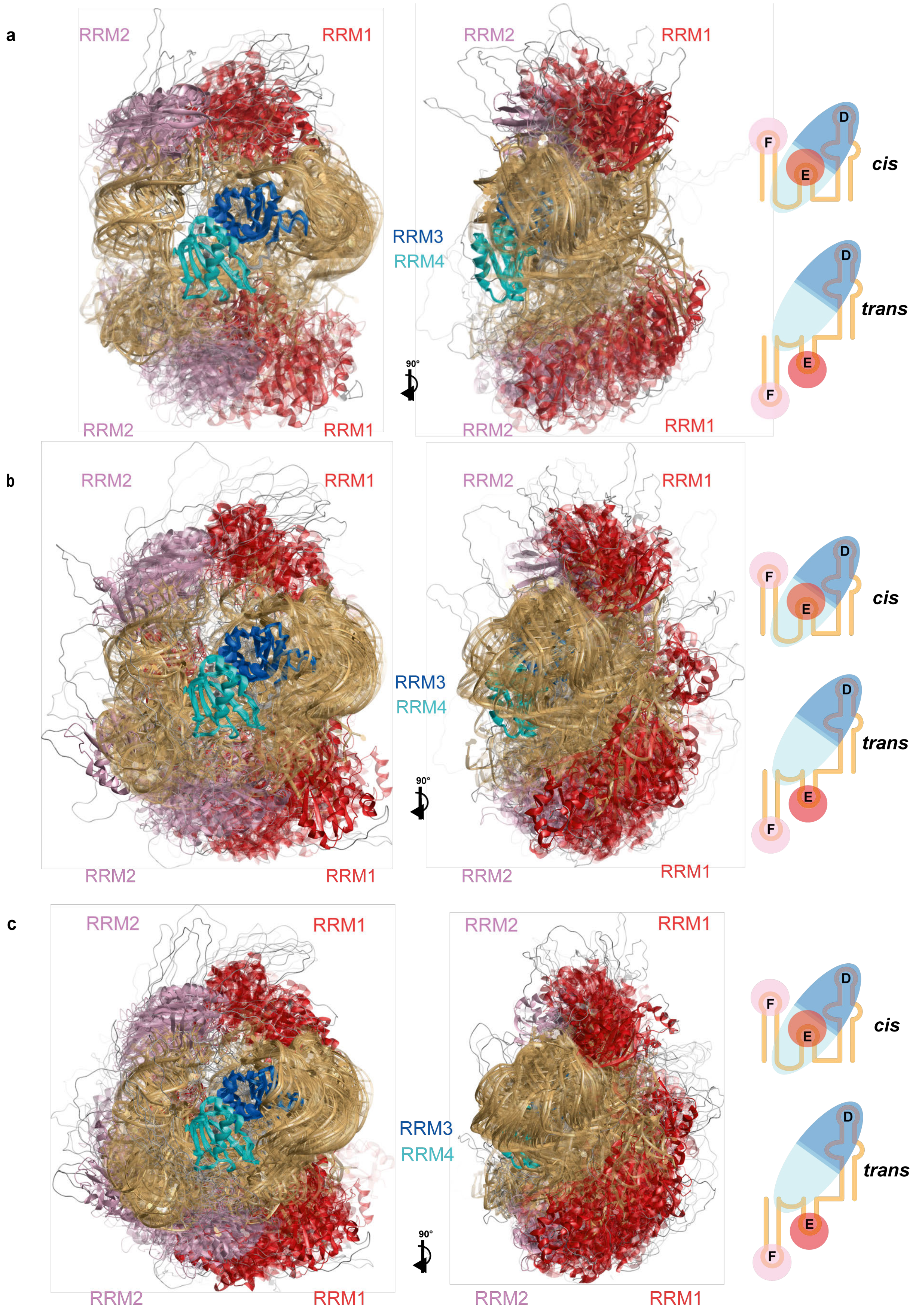

Supplementary Fig. 7. PTBP1/EMCV-IRES-DtoF ensembles generated for testing robustness of modelling. **a** An ensemble obtained in another fit after removing the 25 conformers that were included in the best-fitted integrative ensemble. This validation ensemble has 30 conformers. Fit of the small-angle scattering curves has

deteriorated (χ^2: 15.23) and fit of the DEER distance distributions (geometric average: 0.683) and loss of merit (0.266) have even slightly improved. **b** Structural ensemble of the PTBP1/EMCV-IRES-DtoF complex generated by ensemble reweighting with a non-negative linear least squares approach. This NNLLSQ ensemble has 48 conformers. **c** Structural ensemble of the PTBP1/EMCV-IRES-DtoF complex generated by ensemble fitting only against DEER data. This DEER-only ensemble has a slightly better fit for all the distance distributions with an overlap between experimental and back-calculated distance distributions between 0.614 and 0.933 with a geometric average of 0.783. Structural ensemble in a conformer population-weighted visualization. The 78 models of the ensemble were superimposed on RRM34. Two views related by a vertical rotation of 90° are shown. As in the integrative structural ensemble, SLE and SLF can be positioned in *cis* or *trans* with respect to SLD, illustrated in schemes on the right.

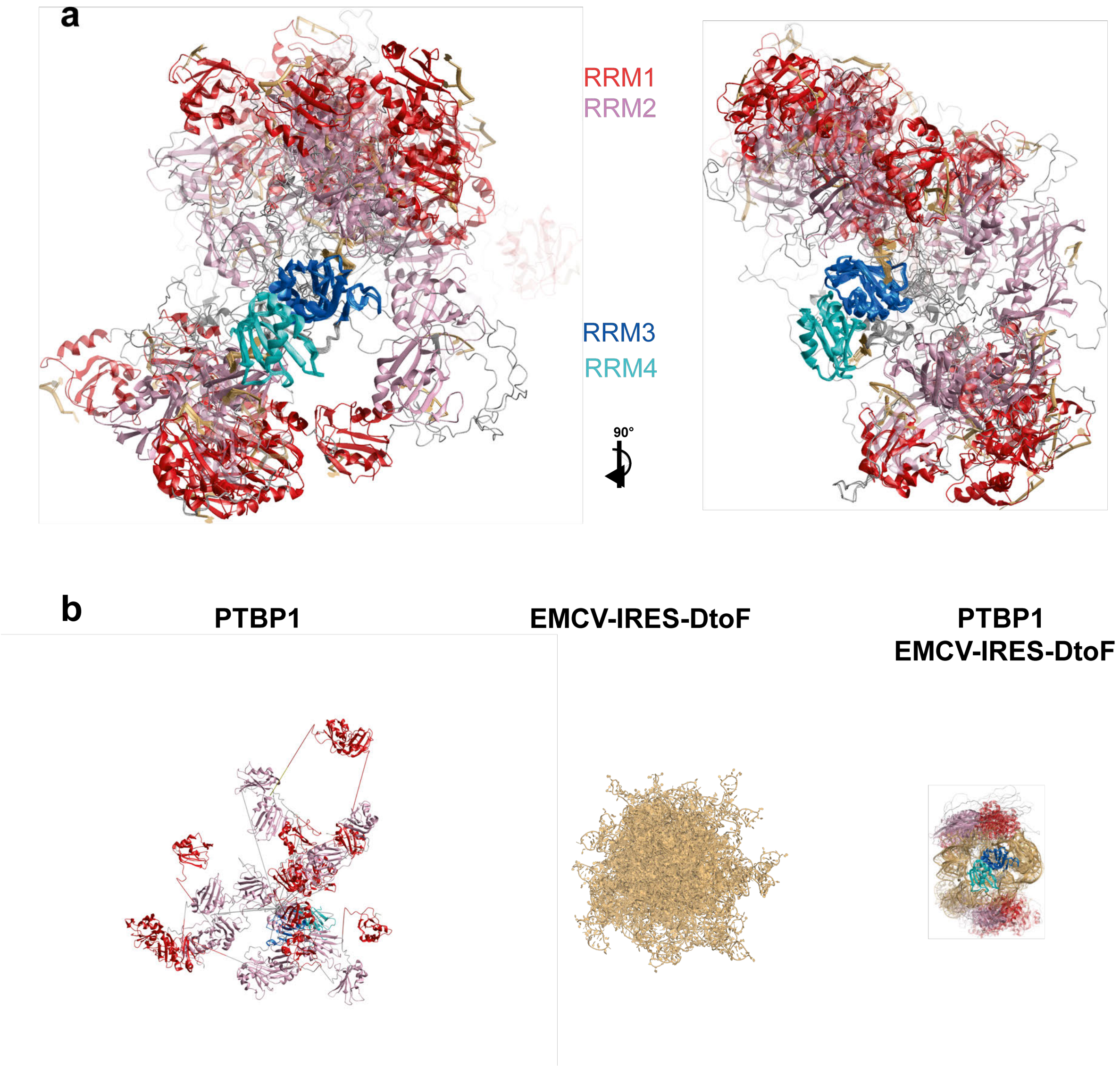

Supplementary Fig. 8. PTBP1 protein-only ensemble obtained with the restraint set for the PTBP1/EMCV-IRES-DtoF complex excluding the SAXS curve and unrestrained component ensembles. a Ensemble of only PTBP1 and RNA binding motifs obtained with the restraint set for the PTBP1/EMCV-IRES-DtoF complex excluding the SAXS curve. This protein-only ensemble has 26 conformers. Without the topological restraints caused by the RNA and without the SAXS curve, a slightly different conformational space is accessible, while the *cis* and *trans* subensembles are still recognizable. **b** Ensemble of free PTBP1 fitted to the SANS curve shown in Fig. 1c of the main text and unrestrained EMCV-IRES-DtoF ensembles (100 conformers) superimposed on RRM34 or SLF, respectively, in comparison to the integrative ensemble of the PTBP1/EMCV-IRES-DtoF

complex. The unrestrained EMCV-IRES-DtoF ensemble fits the SAXS curve of free EMCV-IRES-DtoF with a χ^2 value of 1.295.

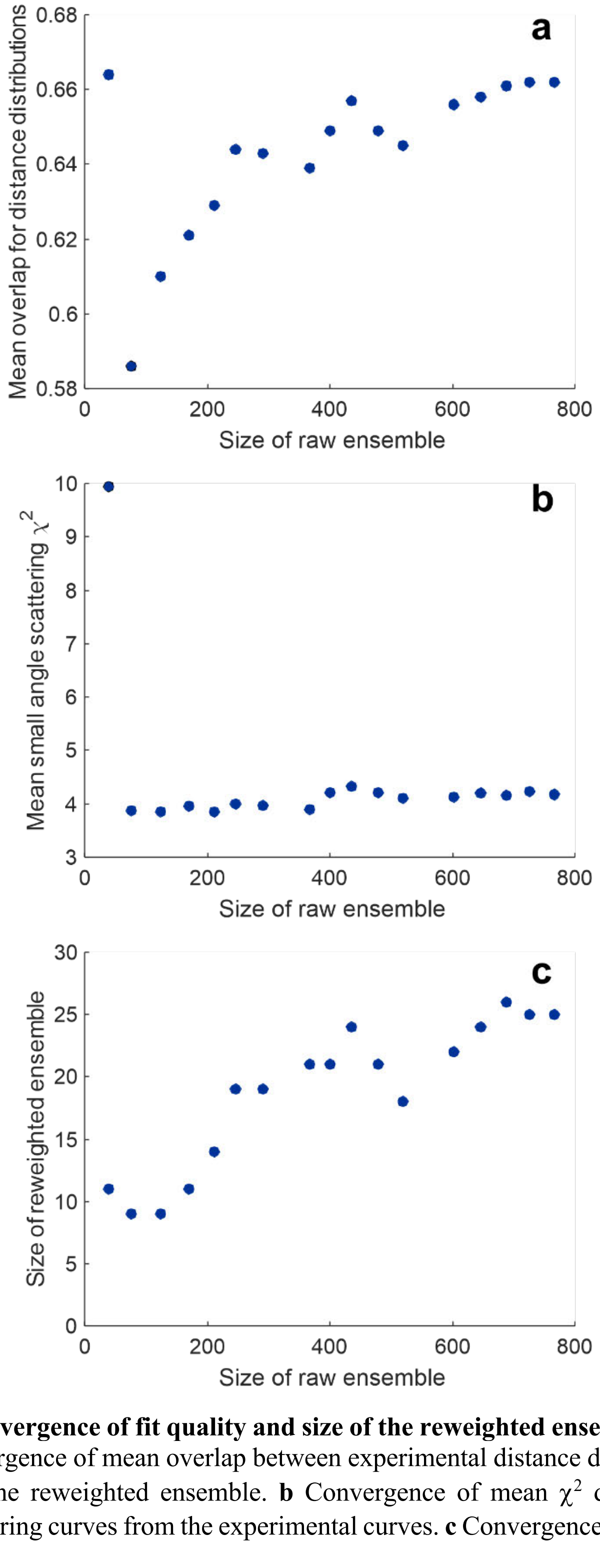

Supplementary Fig. 9. Convergence of fit quality and size of the reweighted ensemble with respect to size of the raw ensemble. a Convergence of mean overlap between experimental distance distributions and distributions and back-calculated from the reweighted ensemble. **b** Convergence of mean χ^2 deviation of the three back-calculated small-angle scattering curves from the experimental curves. **c** Convergence of the size of the reweighted ensemble.

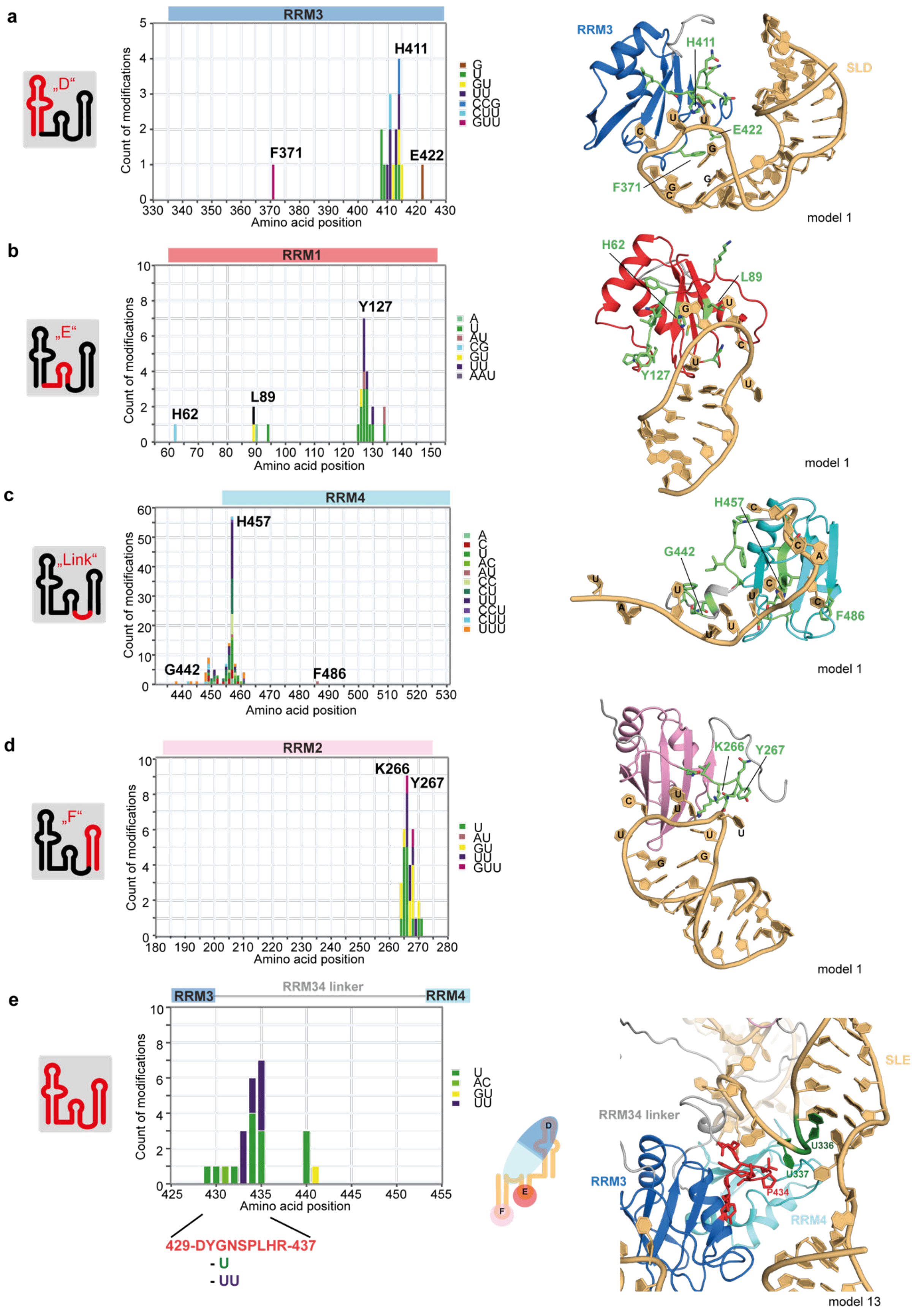
a
RRM3
H411
F371
E422
Count of modifications
Amino acid position
G
U
GU
UU
CCG
CUU
GUU
RRM3
H411
SLD
E422
F371
model 1
b
RRM1
Y127
L89
H62
A
U
AU
CG
GU
UU
AAU
H62
L89
Y127
model 1
c
RRM4
H457
G442
F486
A
C
U
AC
AU
CC
CU
UU
CCU
CUU
UUU
H457
G442
F486
model 1
d
RRM2
K266
Y267
U
AU
GU
UU
GUU
K266
Y267
model 1
e
RRM3
RRM34 linker
RRM4
429-DYGNSPLHR-437
- U
- UU
U
AC
GU
UU
SLE
RRM34 linker
RRM3
U336
U337
P434
RRM4
model 13

Supplementary Fig. 10. Comparison of structural ensemble with updated CLIR-MS/MS data of PTBP1 in complex with segmentally or uniformly isotope labeled RNA. CLIR-MS/MS data were re-analyzed with an updated analysis pipeline. **a**-**d** Cross-link identification detected at each labeling site (D, E, Link, F) for the respective RRM plotted on the sequence of the protein. Colors indicate type of cross-link modification (left). Cross-links shown on the structural models of the corresponding sub-complexes (right). **e** Cross-links identified in the linker between RRM3 and RRM4.

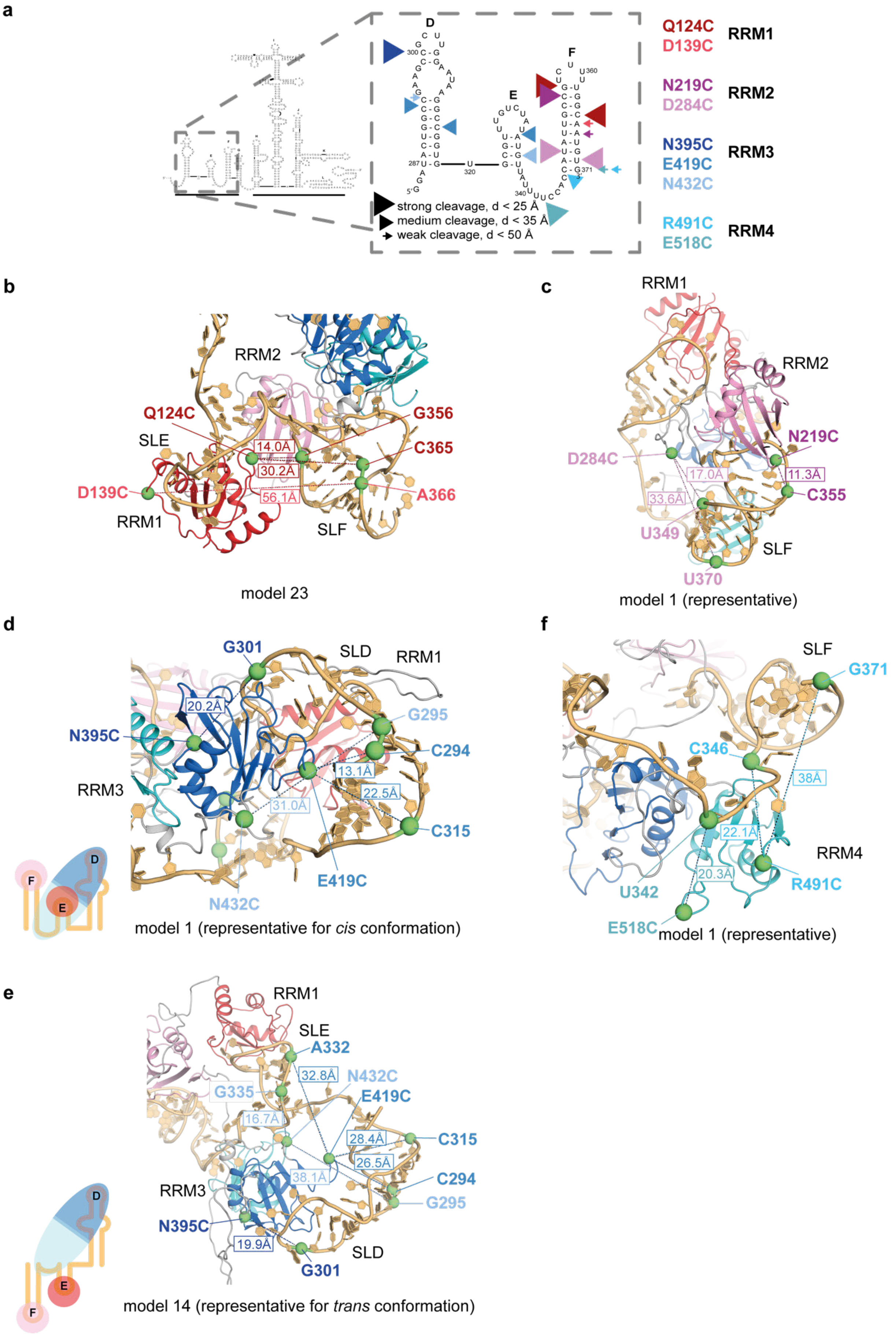

a
D
E
F
strong cleavage, d < 25 Å
medium cleavage, d < 35 Å
weak cleavage, d < 50 Å
Q124C
D139C
RRM1
N219C
D284C
RRM2
N395C
E419C
N432C
RRM3
R491C
E518C
RRM4
b
RRM2
Q124C
SLE
D139C
RRM1
G356
C365
A366
SLF
14.0Å
30.2Å
56.1Å
model 23
c
RRM1
RRM2
D284C
N219C
17.0Å
11.3Å
33.6Å
C355
U349
SLF
U370
model 1 (representative)
d
G301
SLD
RRM1
20.2Å
G295
N395C
C294
13.1Å
22.5Å
31.0Å
RRM3
C315
E419C
N432C
D
F
E
model 1 (representative for cis conformation)
f
SLF
G371
C346
38Å
22.1Å
RRM4
20.3Å
R491C
U342
E518C
model 1 (representative)
e
RRM1
SLE
A332
32.8Å
N432C
G335
E419C
16.7Å
28.4Å
C315
26.5Å
38.1Å
C294
G295
RRM3
N395C
19.9Å
G301
SLD
D
E
F
model 14 (representative for trans conformation)

Supplementary Fig. 11. Comparison of structural ensemble with hydroxyl radical probing data. a Strong, medium and weak cleavages detected for PTBP1 in complex with EMCV-IRES. **b** RRM1 cleavages on SLF are generally not consistent with the mapping of the domain on SLE, however, in a few models (e.g. model 23) residues are close enough to induce a cleavage in SLF. **c** RRM2 cleavages in SLD fit well with the mapping of the domain to this stem-loop and in all the models, the distances are consistent with hydroxyl radical cleavage data. In contrast to previous assumptions, RRM2 does not bind the stem of SLF. **d** RRM3 cleavages on SLD are fully consistent with the mode of binding to SLD in all the models, some cleavages on SLE are rationalized by close distances to SLE in the *trans* conformation. **e** RRM4 cleavages to the LinkEF sequence and the basis of SLF fit well with all models in the ensemble.

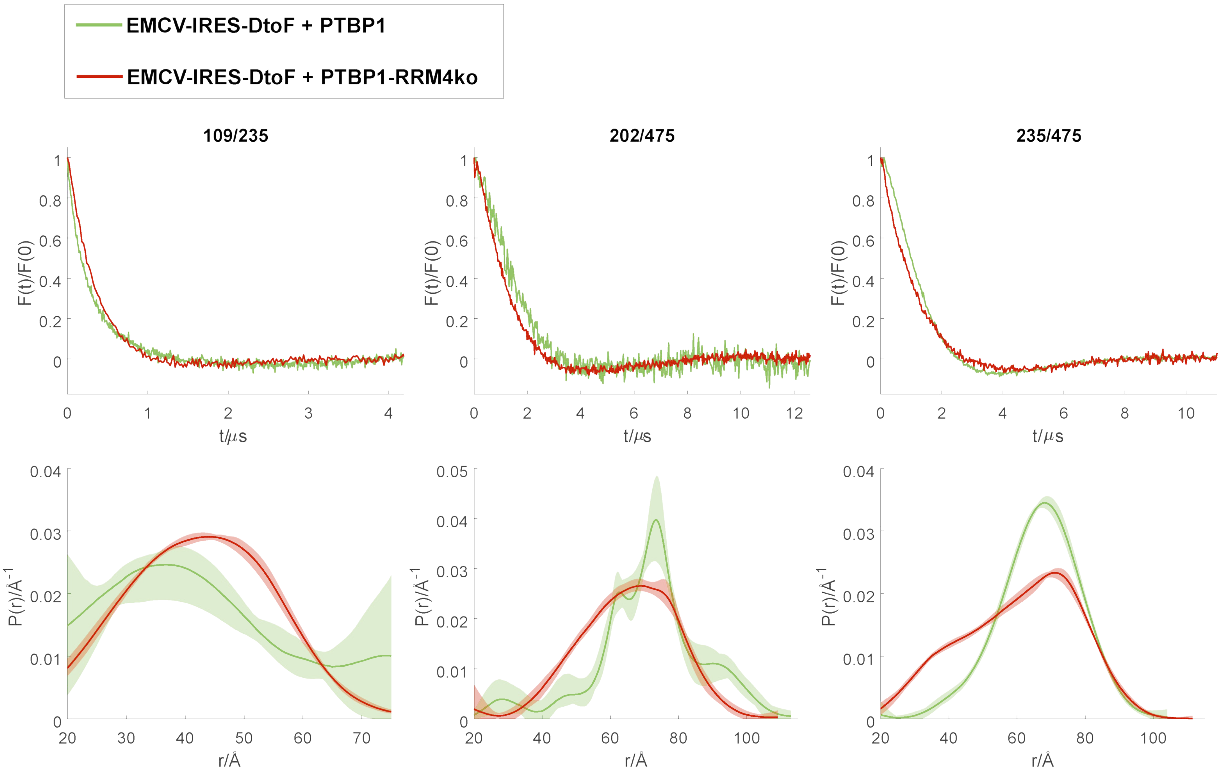

Supplementary Fig. 12. DEER distance measurements of RRM4ko mutants. DEER measurement of WT PTBP1 and construct with mutated binding interface of RRM4 (RRM4ko, red) in complex with EMCV-IRES-DtoF. Three spin-pairs reflecting RRM1-RRM2 (109/235) and RRM2-RRM34 (202/475 and 235/475) distances, respectively. Top panel, form factor and lower panel, distance distribution.

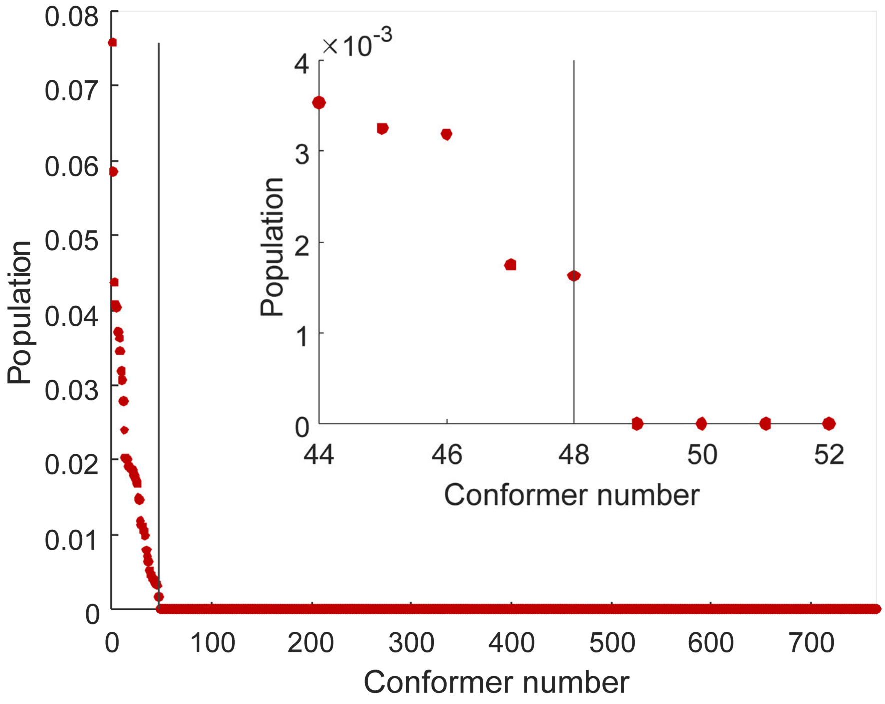

Supplementary Fig. 13. Non-negative linear least squares fitting of 761 populations to 35 distance distributions and 3 small-angle scattering curves turns up only 48 populations that differ significantly from zero. Plotted are populations normalized to a sum of 1 after sorting them in decreasing order. The inset shows the transition from small, but finite populations to populations that are indistinguishable from zero.

Supplementary Table 1. List of cardiovirus sequences used in the alignment and their accession numbers.

1. EMCV-2 (KC310737, KC310738);
2. EMCV-2 (JX257003);
3. MNGV (KX231802);
4. MNGV (MNGPOLY, DQ294633);
5. EMCV-1 (EMCBCG, EMCDG, EMCIAB, DD13364);
6. EMCV-1 (AY296731);
7. EMCV-1 (KF836388);
8. EMCV-1 (EMCPOLYP, DQ517424, DQ464062, KM269482, KJ524643, DQ464063, KF771002, KF293299);
9. EMCV-1 (MH191297);
10. CaV-F1(KY432930);
11. CaV-C1 (NC_038305);
12. CaV-E1 (KY432928);
13. TMEV (EU718733);
14. TMEV (TMENCRF);
15. TMEV (MF416403);
16. TMEV (EU718732);
17. TMEV-1 (TMENCRE, TMEPP, DQ401688);
18. TMEV (EU723238);
19. TMEV-1 (JX443418, TMECG, MK343442, MK343443, KF680264);
20. TMEV (HQ652539);
21. SAFV (MG571790, MG571791, MG571792);
22. SAFV-9 (AB747256);
23. SAFV (FM207487);
24. SAFV (HM142583);
25. SAFV (HM142584);
26. SAFV (FJ464756, FJ464757, FJ464758, FJ464759, JX122400, JX122401, JX122402, JX122403);
27. SAFV-3 (HM181997, HM181998);
28. SAFV (EU681174, EU681175, EU681178, EU681179);
29. SAFV (JX163901);
30. SAFV-10 (AB747257);
31. SAFV (HM142582);
32. SAFV (HQ696920);
33. SAFV (HQ696921);
34. SAFV (GU943518);
35. SAFV (HM142585);
36. SAFV (EU681172, EU681176, JN652231, JN652232, JN652233, JN209931);
37. SAFV (JN209932);
38. SAFV (MH778548);
39. SAFV (NC_010810);
40. SAFV (EU681173, EU681177);
41. SAFV (AB747255);
42. SAFV-5 (FJ463615, AB747258);
43. SAFV-6 (FJ463617);
44. SAFV-3 (HM181996);
45. SAFV (FJ463616).

Supplementary Table 2. Molecular weight of components, experimental radii of gyration (Rg), maximal distance (Dmax).

	MW (kDa), calculated	Rg (Å)	Dmax (Å)
EMCV-DtoF	28	33.41	129.00
PTBP1	56	46.20	168.20
PTBP1-EMCV-DtoF-complex	84	45.03	138.75

Supplementary Table 3. Distance distribution measurements of doubly-labeled PTBP1 in complex with EMCV-IRES-DtoF. The Q-band DEER measurements probe a range from about 15 Å to about 100 Å. The upper limit varies between samples and can be inferred from Supplementary Figure 5.

Double mutant	Mean Distance / Å	Standard deviation / Å	Spin Label Type
**Inter-RRM and RRM-loop data**			
71 -- 202	49 ± 3	19 ± 1	IAP
71 -- 205	46 ± 2	19 ± 2	MTSL
71 -- 235	34 ± 2	12 ± 1	MTSL
71 -- 240	48 ± 4	21 ± 2	MTSL
71 -- 388	50 ± 3	23 ± 2	IAP
71 -- 475	68 ± 3	14 ± 3	MTSL
80 -- 205	45 ± 1	17 ± 1	MTSL
80 -- 235	42 ± 2	18 ± 2	MTSL
80 -- 352	60 ± 2	16 ± 1	MTSL
80 -- 388	61 ± 6	28 ± 8	MTSL
80 -- 475	71 ± 1	11 ± 2	MTSL
109 -- 235	41 ± 2	16 ± 2	IAP
109 -- 240	49 ± 3	17 ± 1	MTSL
109 -- 388	70 ± 3	17 ± 2	MTSL
109 -- 475	80 ± 1	10 ± 1	MTSL
116 -- 240	46 ± 5	25 ± 8	MTSL
152 -- 235	33 ± 1	12 ± 1	MTSL
152 -- 388	43 ± 5	25 ± 9	MTSL
152 -- 475	73 ± 9	24 ± 5	MTSL
235 -- 156	32 ± 5	14 ± 2	IAP
202 -- 388	59 ± 2	17 ± 2	IAP
202 -- 475	71 ± 1	16 ± 2	MTSL
205 -- 352	59 ± 1	8 ± 3	MTSL
205 -- 388	53 ± 1	17 ± 1	MTSL
235 -- 288	48 ± 1	7 ± 2	MTSL
235 -- 315	45 ± 3	22 ± 5	MTSL
235 -- 327	50 ± 2	18 ± 3	MTSL
235 -- 388	59 ± 1	17 ± 2	IAP
235 -- 475	67 ± 1	12 ± 1	IAP
235 -- 500	62 ± 3	12 ± 3	MTSL
388 -- 288	45 ± 1	18 ± 5	MTSL
388 -- 315	30 ± 3	13 ± 2	MTSL
352 -- 327	42 ± 2	14 ± 1	MTSL
388 -- 327	25 ± 1	7 ± 1	MTSL
475 -- 327	53 ± 1	12 ± 1	MTSL

**Intra-RRM data**			
71--109	32 ± 1	3 ± 2	MTSL
205-240	20 ± 1	6 ± 1	MTSL
388--475	46 ± 3	11 ± 4	IAP

Supplementary Table 4. Populations in percent of the 25 conformers of the main ensemble.

**Conformer**	**Percentage**
1	5.0
2	14.9
3	1.6
4	2.8
5	3.2
6	9.1
7	5.2
8	4.4
9	1.8
10	9.2
11	0.2
12	2.3
13	11.5
14	1.0
15	2.2
16	0.4
17	4.7
18	4.1
19	5.4
20	1.1
21	1.9
22	1.7
23	2.1
24	2.7
25	1.6

Supplementary Table 5. Fit quality for the various ensembles. Reported are the geometric average overlap *o* of simulated and experiments distance distributions and χ^2 values of all small-angle scattering curves.

**Ensemble**	***o***	χ^2_{SAXS}	$\chi^2_{\mathrm{SANS,1.2\,m}}$	$\chi^2_{\mathrm{SANS,4\,m}}$
main	0.661	5.08	5.92	0.56
validation	0.683	6.43	8.28	0.52
NNLLSQ	0.755	20.2	10.9	0.51
DEER-only	0.783	35.1	10.8	0.51
raw	0.715	38.9	10.6	0.51

Supplementary Table 6. Overlap of pseudo-electron density among the various ensembles.

Ensemble	main	validation	NNLLSQ	DEER-only
**main**	1	0.790	0.731	0.684
**validation**	0.790	1	0.759	0.712
**NNLLSQ**	0.731	0.759	1	0.866
**DEER-only**	0.684	0.712	0.866	1

Supplementary Table 7. Primer list.

Positions	Primer sequence
T71C-fwd	5'-CCATCGACGTCTGCGAGGGGGAAGTC-3'
T71C-rev	5'-GACTTCCCCCTCGCAGACGTCGATGG-3'
T109C-fwd	5'-GAGGCTGCCAACTGCATGGTGAACTAC-3'
T109C-rev	5' GTAGTTCACCATGCAGTTGGCAGCCTC 3'
S205C-fwd	5'-GCA CCA GAT TTT CTG CAA GTT CGG CAC AG-3'
S205C-rev	5'-CTG TGC CGA ACT TGC AGA AAA TCT GGT GC-3'
S240C-fwd	5'-CGC CAA GCT GTG CCT GGA CGG GCA G-3'
S240C-rev	5'-CTG CCC GTC CAG GCA CAG CTT GGC G-3'
Q388C-fwd	5' GACGGCAACCAGGCCTGCCTGGCCATGAGCCAC 3'
Q388C-rev	5' GTGGCTCATGGCCAGGCAGGCCTGGTTGCCGTC 3'
S475C-fwd	5'-GGTCCTGTTTTGCAGCAATGGGG-3'
S475C-rev	5'-CCCCATTGCTGCAAAACAGGACC-3'
Y361F-fwd	5’-TTT TCG GCG TCT TCG GTG ACG TGC A-3’
Y361F-rev	5’- TGC ACG TCA CCG AAG ACG CCG AAA A-3’
Y430F-fwd	5’- TGA CCA AGG ACT TCG GCA ACT CAC C-3’
Y430F-rev	5’- GGT GAG TTG CCG AAG TCC TTG GTC A-3’
S392C-fwd	5'-CAG CTG GCC ATG TGC CAC CTG AAC G-3'
S392C-rev	5'-CGT TCA GGT GGC ACA TGG CCA GCT G-3'
E468C-fwd	5'-CCC TCA GTC TCC GAG TGC GAT CTC AAG GTC CTG T-3'
E468C-rev	5'-ACA GGA CCT TGA GAT CGC ACT CGG AGA CTG AGG G-3'
V472C-fwd	5'-CGA GGA GGA TCT CAA GTG CCT GTT TTC CAG CAA-3'
V472C-rev	5'-TTG CTG GAA AAC AGG CAC TTG AGA TCC TCC TCG-3'

Supplementary Table 8: SAS data collection parameters

SANS

Source, instrument and description or reference	SINQ Paul Scherrer Institute Switzerland, SANS-I beamline
Wavelength (Å or nm)	0.45 nm
Beam geometry (size, sample-to-detector distance)	sample-to-detector distance: 2 m and 11 m<br>collimation: 3 m and 11 m<br>cross section collimator 50 x 50 cm^2
q-measurement range ($Å^{-1}$ or nm^{-1})	0.08 nm^{-1}<q<3 nm^{-1}
Absolute scaling method	1mm H2O sample that was calibrated against polymer standard for wavelenght dependency of g(lambda) factor of the detector of SANS-1
Basis for normalization to constant counts	sum of counts on monitor 1
Method for monitoring radiation damage, X-ray dose where relevant	NA
Exposure time, number of exposures	variable, 1000000 counts on Monitor 1
Sample configuration including path length and flow rate where relevant	sample depth 1 mm, effective sample diameter: 0.8 x 1.5 cm^2
Sample temperature (ºC)	36 °C

SAXS

Source, instrument and description or reference	Rigaku MicroMax-002 microfocused beam (4 kW, 45 kV, 0.88 mA)
Wavelength (Å or nm)	λ = 1.5418 Å
Beam geometry (size, sample-to-detector distance)	Three pinholes (0.4, 0.3, and 0.8 mm) collimators, detector distance 2m
q-measurement range ($Å^{-1}$ or nm^{-1})	0.1 nm-1< q < 4 nm-1
Absolute scaling method	Back ground and solvent correction, absolute scaling by water intensity
Basis for normalization to constant counts	Rigaku standard normalization procedure.
Method for monitoring radiation damage, X-ray dose where relevant	Not observed
Exposure time, number of exposures	3600 s
Sample configuration including path length and flow rate where relevant	Sample configurated with the scattered intensity standard Rigaku method
Sample temperature (ºC)	23° C

Supplementary Table 9. Radii of gyration of the various ensembles and their standard deviations. The radius of gyration for free PTBP1 according to analysis of the SAS data shown in Fig. 1 is 42.7 Å.

Ensemble	Radius of gyration / Å	Standard deviation / Å
main	31.3	1.2
validation	31.7	1.0
NNLLSQ	32.4	1.1
DEER-only	32.5	1.1
raw	32.6	1.2

Supplementary Table 10. Population-weighted structure quality report for the main ensemble (MolProbity).

Quality measure	Value
Clash score	164.5
Rotamer outliers (%)	2.3
Favored rotamers (%)	93.7
Ramachandran outliers (%)	2.4
Ramachandran allowed (%)	7.6
Ramachandran favored (%)	90.0
Bad bonds (%)	< 0.1
Band angles (%)	0.3
MolProbity rank (%)	9
