# Supplementary material for: Integrative solution structure of PTBP1-IRES complex reveals strong compaction and ordering with residual conformational flexibility
Source: Nat Commun. 2023 Oct 13;14:6429. doi: 10.1038/s41467-023-42012-z (PMC10576089; doi:10.1038/s41467-023-42012-z)
Supplement: Supplementary file 2 — Reporting Summary [file 41467_2023_42012_MOESM2_ESM.pdf]

nature portfolio

Corresponding author(s): Frederic Allain, Gunnar Jeschke and Maxim Yulikov

Last updated by author(s): Sep 14, 2023

Reporting Summary

Nature Portfolio wishes to improve the reproducibility of the work that we publish. This form provides structure for consistency and transparency in reporting. For further information on Nature Portfolio policies, see our Editorial Policies and the Editorial Policy Checklist.

Statistics

For all statistical analyses, confirm that the following items are present in the figure legend, table legend, main text, or Methods section.

n/a	Confirmed	
☐	☒	The exact sample size (*n*) for each experimental group/condition, given as a discrete number and unit of measurement
☐	☒	A statement on whether measurements were taken from distinct samples or whether the same sample was measured repeatedly
☒	☐	The statistical test(s) used AND whether they are one- or two-sided<br>*Only common tests should be described solely by name; describe more complex techniques in the Methods section.*
☒	☐	A description of all covariates tested
☒	☐	A description of any assumptions or corrections, such as tests of normality and adjustment for multiple comparisons
☐	☒	A full description of the statistical parameters including central tendency (e.g. means) or other basic estimates (e.g. regression coefficient) AND variation (e.g. standard deviation) or associated estimates of uncertainty (e.g. confidence intervals)
☒	☐	For null hypothesis testing, the test statistic (e.g. *F*, *t*, *r*) with confidence intervals, effect sizes, degrees of freedom and *P* value noted<br>*Give P values as exact values whenever suitable.*
☒	☐	For Bayesian analysis, information on the choice of priors and Markov chain Monte Carlo settings
☒	☐	For hierarchical and complex designs, identification of the appropriate level for tests and full reporting of outcomes
☒	☐	Estimates of effect sizes (e.g. Cohen's *d*, Pearson's *r*), indicating how they were calculated

Our web collection on statistics for biologists contains articles on many of the points above.

Software and code

Policy information about availability of computer code

Data collection	Data collection MMMx is available at https://github.com/gjeschke/MMMx.
Data analysis	DEER data were analyzed in terms of distances distributions by Deerlab, available through https://github.com/Jeschkelab/Deerlab

For manuscripts utilizing custom algorithms or software that are central to the research but not yet described in published literature, software must be made available to editors and reviewers. We strongly encourage code deposition in a community repository (e.g. GitHub). See the Nature Portfolio guidelines for submitting code & software for further information.

Data

Policy information about availability of data

All manuscripts must include a data availability statement. This statement should provide the following information, where applicable:

- Accession codes, unique identifiers, or web links for publicly available datasets
- A description of any restrictions on data availability
- For clinical datasets or third party data, please ensure that the statement adheres to our policy

As the integrative structure modelling approach is not listed as an accepted experimental method for structure generation by the wwPDB, the structural ensemble have been deposited in Zenodo (DOI: 10.52 81/zenodo.7798970; https://zenodo.org/record/7798970#.ZDVaXvbP0uU). We have also uploaded the DEER and SAS data to Zenodo (DOI: 10.52 81/zenodo.6809478; https://zenodo.org/record/6809478#.YsfWP-xBwUE) Additionally, the MMMx script for ensemble fitting was uploaded to Zenodo under the same DO1. The reanalyzed mass spectrometry proteomics data have been deposited to the ProteomeXchange Consortium {http://proteomecentral.proteomexchange.org) via the PRIDE partner repository with the dataset identifier PXD034894.

Human research participants

Policy information about studies involving human research participants and Sex and Gender in Research.

Reporting on sex and gender	N/A
Population characteristics	N/A
Recruitment	N/A
Ethics oversight	N/A

Note that full information on the approval of the study protocol must also be provided in the manuscript.

Field-specific reporting

Please select the one below that is the best fit for your research. If you are not sure, read the appropriate sections before making your selection.

☒ Life sciences ☐ Behavioural & social sciences ☐ Ecological, evolutionary & environmental sciences

For a reference copy of the document with all sections, see nature.com/documents/nr-reporting-summary-flat.pdf

Life sciences study design

All studies must disclose on these points even when the disclosure is negative.

Sample size	No sample size calculation was performed. All experiments were performed at least in triplicate, which is common practice for molecular biology experiments. We consider this sufficient since the observed effect sizes were large in comparison to the variability between replicates.
Data exclusions	No data was excluded.
Replication	All experiments described in this study were successfully reproduced at least three times and n numbers are provided in the figure legends. All experimental parameters required for the reproduction by other labs are provided in the methods section. All attempts at replication were successful.
Randomization	Randomisation was not applicable as samples were not assigned to experimental groups
Blinding	Blinding was not relevant for this study as no subjective rating (e.g manual counting of features) was performed. Quantitative measurements were performed by machines and the effects described in qualitative experiments were obvious and of large size

Reporting for specific materials, systems and methods

We require information from authors about some types of materials, experimental systems and methods used in many studies. Here, indicate whether each material, system or method listed is relevant to your study. If you are not sure if a list item applies to your research, read the appropriate section before selecting a response.

Materials & experimental systems

n/a	Involved in the study	
x		Antibodies
	x	Eukaryotic cell lines
x		Palaeontology and archaeology
x		Animals and other organisms
x		Clinical data
x		Dual use research of concern

Methods

n/a	Involved in the study	
x		ChIP-seq
x		Flow cytometry
x		MRI-based neuroimaging

Eukaryotic cell lines

Policy information about cell lines and Sex and Gender in Research

Cell line source(s)	HEK-293T from ATCC
Authentication	no further authentication was performed
Mycoplasma contamination	no contamination were detected by PCR
Commonly misidentified lines (See ICLAC register)	no commonly misidentified lines were used in this study
